# Anti-Aging Activity and Modes of Action of Compounds from Natural Food Sources

**DOI:** 10.3390/biom13111600

**Published:** 2023-10-31

**Authors:** Lili Song, Shicui Zhang

**Affiliations:** 1 Key Laboratory of Biomedical Materials of Zhangjiakou, College of Lab Medicine, Hebei North University, Zhangjiakou 075000, China; songlili922819@163.com; 2 College of Life and Geographic Sciences, Kashi University, Kashi 844000, China; 3 Xinjiang Key Laboratory of Biological Resources and Ecology of Pamirs Plateau, Kashi 844000, China; 4 Department of Marine Biology, Institute of Evolution & Marine Biodiversity, Ocean University of China, 5 Yushan Road, Qingdao 266003, China

**Keywords:** foods, bioactive compounds, polysaccharides, polyphenols, carotenoids, sterols, terpenoids, vitamins, anti-aging, lifespan

## Abstract

Aging is a natural and inescapable phenomenon characterized by a progressive deterioration of physiological functions, leading to increased vulnerability to chronic diseases and death. With economic and medical development, the elderly population is gradually increasing, which poses a great burden to society, the economy and the medical field. Thus, healthy aging has now become a common aspiration among people over the world. Accumulating evidence indicates that substances that can mediate the deteriorated physiological processes are highly likely to have the potential to prolong lifespan and improve aging-associated diseases. Foods from natural sources are full of bioactive compounds, such as polysaccharides, polyphenols, carotenoids, sterols, terpenoids and vitamins. These bioactive compounds and their derivatives have been shown to be able to delay aging and/or improve aging-associated diseases, thereby prolonging lifespan, via regulation of various physiological processes. Here, we summarize the current understanding of the anti-aging activities of the compounds, polysaccharides, polyphenols, carotenoids, sterols, terpenoids and vitamins from natural food sources, and their modes of action in delaying aging and improving aging-associated diseases. This will certainly provide a reference for further research on the anti-aging effects of bioactive compounds from natural food sources.

## 1. Introduction

**Figure 1:**
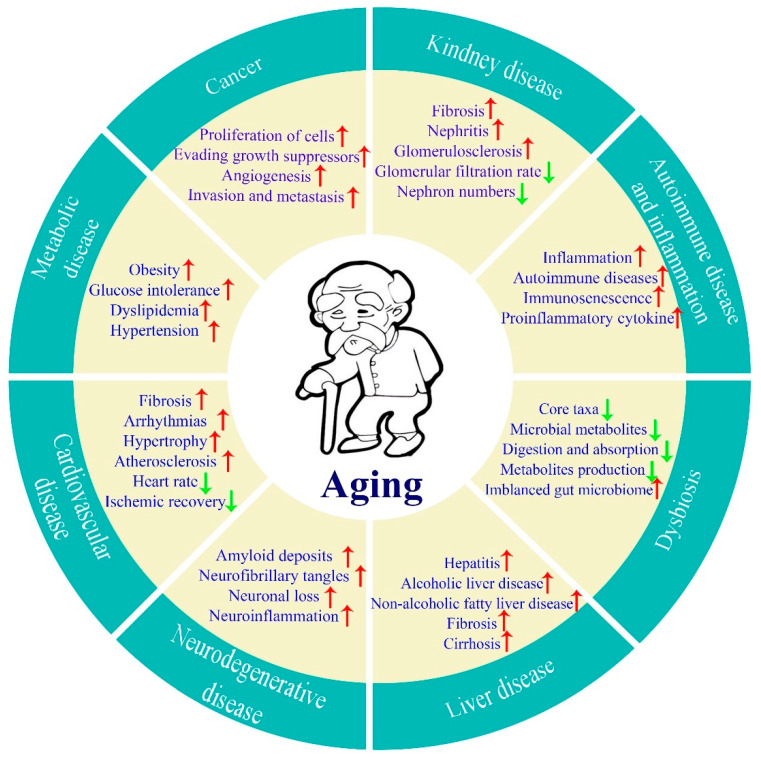
The schematic diagram shows the effects of aging on human health.

We humans all age without exception. Aging is a natural and inescapable phenomenon characterized by a progressive deterioration of physiological functions, leading to increased vulnerability to chronic diseases and death. With economic and medical development, the lifespan of human beings continues to grow throughout the world. It is estimated that there will be more than 795 million people aged 65 years old or over globally by 2023 [1], and this figure will reach 994 million by 2030 and 1.6 billion by 2050, respectively [2]. Aging is usually accompanied by many age-related diseases such as cancer, cardiovascular disease, metabolic disease, kidney disease, liver disease, neurodegenerative disease, autoimmune disease and inflammation, and dysbiosis (Figure 1), as well as other chronic diseases, creating pressure on social health insurances and causing a large economic burden in most countries. Thus, healthy aging has now become a common aspiration among people all over the world.

Aging and anti-aging have attracted curiosity and inspired imagination throughout the history of humankind. In both Eastern and Western civilizations, achieving longevity while postponing aging, or immortality, had long been a fantasy of certain ancient people [3,4]. With the tremendous progress of aging studies over the past three decades, today, anti-aging appears to be no longer a fantasy because the rate of aging can be controlled, at least to some extent, by genetic pathways and biochemical processes conserved in evolution [5,6,7,8]. Numerous theories have been proposed to explain the aging progress, such as mitochondrial free radical theory [9,10], telomere theory [11,12], DNA damage theory [13] and error theory [14]. Broadly speaking, aging theories can be categorized into two major groups: (1) program theory, which suggests that aging is not a random occurrence but instead is genetically programmed in our genetic information, and (2) wear-and-tear theory, proposed by Weismann in 1889 [15], which proposes that aging occurs when cells and tissues are worn down by risk factors over the years. Whatever the theory, aging is generally accompanied by the impairment of various physiological processes such as mitochondrial dysfunction, decline in immunity, slowing down of basal metabolism, imbalance of apoptosis and autophagy, alteration of intestinal flora, reduction in activities of antioxidant enzymes, cellular senescence, and so on [16,17,18,19]. In accordance, substances that can mediate the physiological processes above are highly likely to have the potential to prolong lifespan and improve aging-associated diseases.

Foods from natural sources are full of bioactive compounds such as polysaccharides, polyphenols, carotenoids, sterols, terpenoids and vitamins [20,21]. These bioactive compounds and their derivatives have been shown to be able to delay aging and/or improve aging-associated diseases, thereby prolonging lifespan [22,23,24], via the regulation of various physiological processes, including antioxidant reactions, anti-inflammatory response, immunity, telomere activation, anti-apoptosis and mitochondrial repair, etc. [25,26]. Below, we will discuss the state-of-the-art understanding of the anti-aging activities of the compounds, polysaccharides, polyphenols, carotenoids, sterols, terpenoids and vitamins from natural food sources, and their modes of action in delaying aging and improving aging-associated diseases.

## 2. Anti-Aging Effects of Compounds from Food Sources

Bioactive compounds from natural food sources refer to the extra-nutritional constituents that are typically present in small quantities in foods. Many bioactive compounds have been discovered. These compounds, including polysaccharides, polyphenols, carotenoids, sterols, terpenoids and vitamins, are being intensively studied to evaluate their effects on health, and many of them appear to have anti-aging activities.

### 2.1. Polysaccharides

Polysaccharides are macromolecules consisting of tens to thousands of monosaccharides that are linked by α-1,4, β-1,3, α-1,6 or β-1,6 glycosidic bonds. It has been shown that the polysaccharides with β-(1,3)-D-glucan main-chain structure usually have important bioactive activity [27]. There exist two groups of polysaccharides, homopolysaccharide and heteropolysaccharide, with the former composed of the same monosaccharide, and the latter two or more different monosaccharides. Polysaccharides occur widely present in plants, algae, fungi and bacteria. Many studies have demonstrated that some polysaccharides and their derivatives, extracted from natural food sources, show various pharmacological effects, such as anti-diabetic, anti-tumor, cholesterol-lowering, wound-healing, anti-inflammatory, antioxidative, immune-modulatory and even anti-aging activities [28,29,30]. In addition, polysaccharides have unique advantages in terms of side effects and long-term use due to their low cytotoxicity, which is expected to contribute to the development of novel anti-aging drugs or supplements [30]. Here, we describe the anti-aging activity of polysaccharides from food sources and their modes of action (Table 1).

#### 2.1.1. Plant Polysaccharides

Many studies have demonstrated that plant polysaccharides often display anti-aging effects. Chinese wolfberry *Lycium barbarum* serves as both medicine and food. *L. barbarum* polysaccharide (LBP), the main bioactive substance of wolfberry, was shown to be able to prolong the lifespan of the fruit fly *Drosophila melanogaster* and the nematode *Caenorhabditis elegans* [31,32], and to reverse the estrogen deficiency-induced learning and memory impairments of ovariectomized mice [33]. Moreover, LBP was found to partially protect against UVB-induced photodamage [34] as well as the PM2.5-induced mitophagy and mitochondrial damage of the human keratinocyte HaCaT cells [35], showing anti-skin-aging activity. The edible and medicinal ginseng *Chuanminshen violaceum* polysaccharides (CVPs) were also found to increase the body weight and spleen index of D-galactose (D-gal)-induced-aging mice, suggesting the presence of anti-aging activity [37], and the polysaccharides from ginsenoside residues (GRPs) were found to extend the lifespan of *C. elegans* [38]. Similarly, sweet tea *Rubus suavissmus* polysaccharides (STP-60a) were demonstrated to increase the mean lifespan of *C. elegans*, reduce the lipofuscin (LF) level and improve the survival rate of worms under heat stress and oxidative stress [39].

#### 2.1.2. Algal Polysaccharides

Edible marine algae are rich in bioactive polysaccharides that are widely used in the food and chemical industries as well as in medicine and healthcare due to their benefits to health [45,61]. The kelp *Laminaria japonica*, a marine macroalga, is a common and important sea food for human consumption. *L. japonica* polysaccharide (LJP) was shown to possess an anti-aging effect. LJP could enhance the acute oxidation resistance of *C. elegans* when exposed to high temperatures, prolong the lifespan of worms, and decrease the accumulation of malondialdehyde (MDA), LF and reactive oxygen species (ROS) [45]. The laver *Pyropia haitanensis*, a red alga, is another common sea food. The *P. haitanensis* polysaccharide, porphyrin, was able to significantly ameliorate the learning and memory impairment in Alzheimer’s disease (AD)-model mice, to increase choline acetyltransferase (ChAT) activity, and to decrease acetylcholinesterase (AChE) activity in the cortical and hippocampal tissue of AD-model mice [46]. The asparagus *Gracilaria lemaneiformis*, a brown seaweed, is usually eaten as a vegetable. *G. lemaneiformis* polysaccharides (GPs) could extend the mean lifespan of *C. elegans* and significantly increase the reproduction duration of worms [47].

#### 2.1.3. Fungal Polysaccharides

Edible fungi contain rich bioactive components that have both nutritional and medicinal values, thus making them a potent dietary supplement to attenuate aging and prevent age-related diseases in humans [54,62]. Increasing evidence has shown that polysaccharides are one of the main bioactive components that exhibit anti-aging activity in edible fungi [54,63]. For example, the polysaccharides derived from the bolete *Lanmaoa asiatica* (FLAPs) and the yellow mushroom *Hohenbuehelia serotina* (FHSPs), and the β-glucans derived from the maitake *Grifola frondosa* (GFGs) and the monkey head mushroom *Hericium erinaceus* (HEGs), were all able to prolong the lifespans of *C. elegans* [51] and *D. melanogaster* [52], respectively. The intracellular zinc polysaccharides (IZPs) of *G. frondosa* not only decreased the MDA content but also ameliorated the anile condition of D-gal-induced-aging mice [53]. It was also shown that the sulfated polysaccharides of the golden mushroom *Flammulina velutipes* (SFPs) reversed the D-gal-induced weight loss and tissue damage of aging mice [54], and the polysaccharides of the common cultivated mushroom *Agaricus bisporus* (WMPs) improved the locomotor activity and spatial and recognition memory of the aging mice [55]. Interestingly, WMPs extracted by different methods such as acidic-extraction (AcAPs) and enzyme-assisted extraction (EnAPs) all displayed potential anti-aging effects against D-gal-induced-aging diseases on the liver, kidney, brain and skin [56,57].

### 2.2. Polyphenols

Polyphenols are the secondary metabolites of plants, characterized by at least two phenyl rings and one or more hydroxyl substituents [64]. Based on whether their chemical structure contains sugar or not, and whether it is hydrolyzed or condensed, polyphenols are usually divided into five groups: phenolic acids, flavonoids, stilbenes, lignans and tannins [65]. Polyphenols are found in high quantities in beverages such as tea and red wine, in vegetables such as red pepper, eggplant, garlic and carrot, and in fruits such as grape and mulberry [66,67]. Marine algae also contain rich polyphenols [68]. Previous studies have shown that polyphenols possess multiple biological effects, including antioxidant, anti-aging, anti-inflammatory, neuroprotective, anti-cancer and anti-diabetic activities [69,70,71,72]. For example, walnuts and their by-products, rich in phenolic components, were shown to decrease the levels of ROS and inflammatory cytokines, modulate the Nrf2/EpRE, PI3K/Akt/mTOR and NF-κB signaling pathways, prevent mitochondrial dysfunction, and regulate energy homeostasis, thus presenting anti-aging potential [73]. Here, we describe the anti-aging activity of polyphenols, including phenolic acids, flavonoids, stilbenes, lignans and tannins from natural food sources, and their possible anti-aging mechanisms (Table 2).

#### 2.2.1. Phenolic Acids

Phenolic acids contain an aromatic benzene ring and a carboxylic acid group and mainly exist in wine, sorghum, dried dates, blackberries, apple juice, olives, chicory and black tea. Phenolic acids are subdivided into hydroxybenzoic acid and hydroxycinnamic acid [97]. Previous studies have reported that phenolic acids display prominent anti-aging effects. Chlorogenic acid (CGA), a predominant class of phenolic acids extracted from coffee and tea, could extend the lifespan of *C. elegans* by up to 20.1%, delay the age-related decline of body movement, and improve stress resistance. Moreover, CGA activated the Forkhead box O (FoxO) transcription factor DAF-16, heat shock transcription factor-1 (HSF-1), SKiNhead-1 (SKN-1), and hypoxia-inducible factor-1 (HIF-1), and thus extended the lifespan of *C. elegans* mainly via DAF-16 in the insulin/IGF-1 signaling (IIS) pathway [74]. Similarly, p-Coumaric acid, a kind of hydroxycinnamic acid abundant in peanuts, carrots, and garlic, could increase the survival of *C. elegans* under oxidative and osmosis-stressed conditions. In addition, treatment with p-Coumaric acid could result in a significant reduction of intercellular ROS levels and a marked increase in antioxidant capacity through the regulation of skn-1, an ortholog of the nuclear factor erythroid 2-related factor 2 (Nrf2) transcriptional factor [75].

#### 2.2.2. Flavonoids

Flavonoids are subdivided into flavonols, flavanones, isoflavones, flavones, anthocyanins, and flavan-3-ols according to their chemical structure and have all been shown to possess anti-aging activity. Quercetin, belonging to the flavonols, has been demonstrated to have a positive effect on longevity and stress resistance in various animal models. It was shown that feeding with quercetin prolonged lifespan, suppressed age-related motility retardation, improved motility recovery after heat stress, and reduced the production of both intercellular and mitochondrial ROS in *C. elegans* through the modulation of the insulin-like and mitogen-activated protein kinase (MAPK) pathways [77]. It was also shown that a diet containing quercetin or rutin diminished several early signs of AD-model mice, and the administration of quercetin or rutin increased the glutathione/oxidized glutathione (GSH/GSSG) ratio, diminished the MDA level, and favored the enzyme antioxidant capacity. Moreover, exposure to quercetin or rutin could diminish amyloid precursor protein (APP) synthesis, β-site APP-cleaving enzyme 1 (BACE1) activity, and the expression of the inflammatory markers interleukin-1β (IL-1β) and interferon-γ (IFN-γ) in AD mice [79].

Apigenin, luteolin and genkwanin are other important classes of flavonoids. They were found to possess antioxidant and anti-inflammatory properties capable of inhibiting ROS production and carcinogenesis and modulating the activity of the enzymes involved in neurological function, thereby contributing to anti-aging action [98,99,100]. For example, apigenin was shown to reduce ROS and MDA levels via enhancing activities of the antioxidant enzymes catalase (CAT), superoxide dismutase (SOD) and glutathione peroxidase (GPX), as well as upregulating the levels of antioxidant response proteins, such as Nrf2 and AMPK [98]. An in-vitro study indicated that apigenin prevented the injury response of lipopolysaccharide (LPS)-stimulated RAW 264.7 macrophage cells by enhancing the reduction of NO. Apigenin also decreased the levels of pro-inflammatory cytokines TNF-α, IL-18, and IL-6; downregulated the expression of cyclooxygenase (COX-2) and inducible nitric oxide synthase (iNOS); and reduced intracellular ROS production [98]. In addition, apigenin was able to inhibit the activity of intracellular cell-adhesion molecules (ICAMS), monocyte inflammatory protein (MIP-1α), and monocyte chemotactic protein (MCP-1α) inhibitors induced by LPS, resulting in an anti-inflammatory response [101,102].

Anthocyanins are found in a wide variety of colored vegetables, fruits, and cereal, especially in various berry fruits such as bilberries, blueberries, blackberries, blackcurrants, chokeberries, strawberries, and elderberries [80,103]. It was found that anthocyanin extracted from purple wheat extended the mean lifespan of wild-type nematodes and *mev-1* (*hn1*) mutants, which are sensitive to oxidative stress, by up to 10.5 and 9.2%, respectively, through the activation of DAF-16/FoxO transcription factors [80]. Anthocyanins from the honeysuckle *Lonicera pallasii* were also shown to prolong the median and maximum lifespan of the fruit fly by up to 8% and enhance the integrity of the intestinal barrier. Interestingly, all these effects were accompanied by the increased expression of *Hif1* (pro-longevity gene) and decreased expression of *Keap1* (anti-longevity gene). RNA interference-mediated experiments indicated that the positive effects inspired by anthocyanin administration were associated with *Sirt6* activation [81].

#### 2.2.3. Stilbenes

The most important representative of stilbene compounds is resveratrol, which is mainly present in grape peels, grape seeds, blueberries, peanuts, red wine and some traditional Chinese herbal medicines such as rhubarb *Rheum palmatum* and giant knotweed *Polygonum cuspidatum* [104,105,106]. Resveratrol is usually recommended as a dietary supplement to maintain redox balance and delay aging [107]. Previous studies have shown that resveratrol could extend the lifespans of various organisms, including yeast, nematodes, fruit flies, Mexican fruit flies, turquoise killifish and mice [72]. Resveratrol was revealed to prolong the lifespan of both male and female adult fruit flies through scavenging ROS and neuroprotection [83]. Resveratrol also prolonged the lifespan, improved the cognitive ability, and attenuated the aging-related histological markers of aged annual fish *Nothobranchius guentheri* (*N. guentheri*) [84], and delayed ovarian aging in *N. guentheri* by alleviating inflammation and endoplasmic reticulum (ER) stress through Sirt1/Nrf2 [85]. Interestingly, resveratrol not only extended the lifespan but also delayed the worsening of the motor phenotype of HtrA2 knockout mice [86].

#### 2.2.4. Lignans

Lignans, phenolic dimers possessing a 2,3-dibenzylbutane structure, are found in legumes, grains, seeds and vegetable oils. Sesamin is a major lignan constituent of sesame and has anti-aging activity. It was reported that sesamin could prolong the lifespan of nematodes and reduce amyloid-β toxicity [91]. Sesamin was also able to extend the lifespan of the *mev-1* mutant nematodes that produced abundant superoxide anions [92]. Moreover, sesamin was capable of making nematodes more resistant to infection by the pathogen *Legionella pneumophila* and more resistant to oxidative stressors such as paraquat and H_2_O_2_ treatment [92].

#### 2.2.5. Tannins

Tannic acid (TA) and ellagic acid (EA) are two common tannins with anti-aging effects. It was shown that exposure to low concentrations of TA exhibited potent life-prolonging properties in *C. elegans* and enhanced both thermal-stress resistance and oxidative-stress resistance [93]. EA was also able to prolong the lifespan of nematodes [94]. Moreover, oenothein B (OEB), a dimeric hydrolyzable tannin of macrocyclic structure [95], and pentagalloyl glucose (PGG), a xanthoyl tannin [96], both induced the lifespan extension of nematodes through the regulation of the IIS and dietary restriction (DR) pathway and the mitochondrial electron transport chain. Recently, urolithin A, a kind of intestinal microbiota metabolite of ellagitannin-rich foods, was also shown to exhibit anti-aging activities by enhancing stress resistance, reducing mitochondrial function decrease, and inhibiting the age-related decrease in *C. elegans* [42].

### 2.3. Carotenoids

Carotenoids are a group of pigments that mainly exist in fruits (e.g., pineapple and papaya), flowers (e.g., marigold flower and sunflower), vegetables (e.g., tomato and carrot), algae (e.g., microalgae and brown algae) and seafood (e.g., krill and fish). Organisms containing carotenoids may present yellow, orange or red colors. Carotenoids comprise eight isoprene units, resulting in multiple cis- and trans-isomers, with the latter being more abundant in nature [108] (p. 379) and [109] (p. 681). Carotenoids are divided into two groups, carotenes and xanthophylls, on the basis of their chemical structure. Common carotenes include α-carotene, β-carotene, γ-carotene and lycopene, which are highly soluble in organic solvents [108]. Xanthophylls are the oxygenated derivatives of carotenes. Common xanthophylls are astaxanthin, fucoxanthin, lutein, violaxanthin and canthaxanthin, which are soluble in polar solvents and organic solvents [108,109]. Previous studies have shown that carotenoids have multiple biological effects, including immune-system modulation, cell communication, antioxidant activity, anti-aging effects, anti-inflammatory response, anti-angiogenic activity and antiproliferative activity [110,111]. Here, we describe the anti-aging activities of carotenes and xanthophylls from natural food source and their possible anti-aging mechanisms (Table 3).

#### 2.3.1. Carotenes

β-Carotene is an antioxidant that has been shown to be a cancer suppressor and an anti-inflammatory agent in various animal and cell models [126]. Recently, β-carotene has been reported to possess an anti-aging effect [112]. Exposure to β-carotene reduced the activity of SA-β-Gal, the production of P21, P16 and P53, and levels of pro-inflammatory factors such as IL-1β, IL-6, and tumor necrosis factor-α (TNF-α), accompanied by the downregulation of nuclear factor-kappaB (NF-κB) phosphorylation level, in the senescent mesenchymal stem cells (MSCs) model induced by H_2_O_2_. Exposure to β-carotene also reduced the levels of ROS and MDA but increased the level of SOD. Moreover, exposure to β-carotene not only enhanced learning and memory ability, alleviated anxiety, and improved the physical fitness of aged Y-maze mice, but it also slowed down the aging of tissues and organs and diminished the level of tissue and organ fibrosis. Furthermore, a prospective study of 960 participants of the Memory and Aging Project indicated that in older adults (over 60 years old), the intake of dark green vegetables rich in β-carotene, α-tocopherol, and phylloquinone benefited certain domains of cognition, including learning and memory [127]. All these facts indicate that β-carotene has anti-aging effects. It appears that β-carotene exerts the anti-aging effect through the regulation of the lysine acetyltransferase 7 (KAT7)-P15 (a cyclin that induces a G1-phase cell-cycle arrest) axis [112].

Lycopene is the primary phytochemical of tomatoes, with a potent anti-aging effect. The administration of lycopene was shown to protect the liver, brain, kidney and skin of adult mice against oxidative stress through the enhancement of the activities of antioxidant enzymes [113]. Lycopene was also found to rescue the reduced antioxidant capacity by promoting the activities of antioxidases and activating the Nrf2/Heme oxygenase-1 (HO-1) pathway in both D-gal-induced and naturally aged ovaries of chickens, as well as to promote cell proliferation and inhibit apoptosis in both D-gal-induced and naturally aged ovaries. In addition, lycopene could alleviate D-gal-induced mitochondrial damage in living granulosa cells [114].

Crocin is a natural carotenoid antioxidant obtained from the stigma of saffron flowers [128]. It has recently been reported that crocin possesses anti-aging properties. Middle-aged rats fed with diets containing crocin displayed a relative decline in body weight gain and a reduction in age-associated serum triglyceride levels. Notably, both age-associated macromolecular damage and a decline in endogenous antioxidants as well as an increase in intracellular calcium concentration in the cerebral cortex were all reversed due to the oral administration of crocin. Crocin also improved acetylcholine content and enhanced mitochondrial function [117].

#### 2.3.2. Xanthophylls

Astaxanthin (ATX), naturally found in krill and the microalgae *Haematococcus*, belongs to a group of xanthophylls with greater antioxidative activity due to the presence of 2 oxygenated groups on each ring structure [129]. ATX has been shown to possess anti-aging activity in both yeast and nematodes [120,130,131,132]. In D-gal-induced-aging rats, ATX was found to be able to decrease MDA content, enhance antioxidative activity, and reduce oxidative stress, ultimately resulting in the repair of damaged liver, due to its ability to activate the Keap1/Nrf2 pathway and suppress the NF-κB pathway [118]. Similarly, ATX administration decreased the levels of oxidative stress markers, including MDA, nitric oxide (NO) and advanced protein oxidation product (APOP), and increased the activities of antioxidant enzymes SOD and CAT in the brains of young and aged mice, thus contributing to the retardation of aging [119].

Canthaxanthin widely exists in marine animals, algae and a few terrestrial plants. It was found that treatment with canthaxanthin decreased the activity of SA-β-Gal, the levels of ROS and MDA, and the expression of pro-inflammatory factors (IL-6, IL-1β, TNF-α), chemokines (CXCL1) and metalloproteinases (MMP-1) but increased the levels of SOD and GSH and the proliferation of the aging-model liver cells (AML12) stressed by H_2_O_2_. An in-vivo study also showed that canthaxanthin could reduce the liver fibrosis area of aged mice, decrease serum ALT and AST levels, and downregulate the expression of TNF-α and IL-1β, exhibiting an anti-aging effect on aged livers [123]. These facts indicate that canthaxanthin exhibits anti-aging activity.

Lutein is a superior antioxidant naturally present in green plants and in the fat of plant-eating animals, egg yolk and the retina. It is known that the exposure of skin to UV radiation (photo-aging) leads to a loss of cell viability, membrane damage, and the deposition of excessive elastotic material. Interestingly, lutein was shown to not only improve the viability and membrane integrity of UV-exposed fibroblasts but also to suppress elastin synthesis by the inhibition of the level of matrix metalloproteinases (MMPs). Thus, lutein has anti-skin-aging activity [124].

### 2.4. Sterols

Sterols are essential components of the membranes of all eukaryotic organisms, including fungi, plants, invertebrates and vertebrates, and play roles in controlling membrane fluidity and permeability. In some plants, sterols have a specific function in cell proliferation, signal transduction and the modulation of the activity of membrane-bound enzymes [133,134]. Sterols also exhibit a wide range of pharmacological effects, including immunomodulatory, hepatoprotective, anti-cancer, antimicrobial, antifungal, anti-inflammatory, cardiotonic and anti-aging activities [135,136,137]. Here, we describe the anti-aging activities of sterols from natural food sources and their possible anti-aging mechanisms (Table 4).

#### 2.4.1. Phytosterols

Plant sterols and their saturated derivatives are called phytosterols. The human body cannot synthesize phytosterols, and we must obtain from the diet [148]. More than 250 phytosterols have already been isolated from dietary and medicinal plants [149]. They are divided into sterols and stanols, representing the unsaturated and saturated molecules, respectively [150]. Stigmasterol, β-sitosterol, campesterol, guggulsterone, diosgenin, sarsasapogenin, physalin A and dioscin are the most common phytosterols found in the diet, including in vegetable oils, whole grains, nuts, fruits, and vegetables [151,152]. Vegetable oils and their products are the richest natural sources of plant sterols [153].

The anti-aging activity of phytosterols has attracted much attention in recent years. It was shown that the phytosterols from the seeds of the dodder *Cuscuta chinensis* decreased ROS levels, increased heat-stress resistance, and extended the lifespan of aged *C. elegans* [153]. Our study demonstrated that diosgenin (DG), a naturally occurring steroidal sapogenin found predominantly in the roots of yam *Dioscorea villosa* and the seeds of fenugreek *Trigonella foenum-graecum*, exhibited conspicuous anti-aging activity. We clearly showed that DG remarkably extended both the mean and maximum lifespans of the aged male *N. guentheri*. DG administration also significantly reduced the accumulation of histological aging biomarkers, LF and SA-β-Gal, and decreased the levels of ROS, protein oxidation and lipid peroxidation in aged fish [138]. β-Sitosterol, abundant in nuts, legumes and olive oil, was able to prevent H_2_O_2_-treated human dermal fibroblast (HDF) and human permanent epidermal (HaCaT) cells from death, and to exert anti-skin-aging activity by the promotion of the biosynthesis of hyaluronic acid (HA) and the enhancement of skin barrier function [139]. Daucosterol palmitate (DSP), a plant steroid from the herb *Alpinia oxyphylla*, could ameliorate Aβ-induced learning and memory impairments and inhibit ROS production in a male Alzheimer’s disease rat model [140].

#### 2.4.2. Animal Sterols

Animal sterols contain cholesterol, vitamin D and steroid hormones. Cholesterol, the main animal sterol, acts as a precursor for the synthesis of steroid hormones, vitamin D, bile acids and oxysterols [154]. Vitamin D has two forms: D3 (cholecalciferol) and D2 (ergocalciferol). Animal sterols are present in many animal-derived products, including the flesh of fish, fish oils, egg yolk, beef liver, chicken liver and veal steak [155].

Vitamin D3 is well known for its activities in maintaining and regulating calcium and phosphorus homeostasis and in modulating both male and female reproductive processes [156,157]. The production of vitamin D declines with age in male rodents, leading to the reduction of sperm count, motility and mating ability. This age-associated decline in vitamin D3 level suggests a connection between vitamin D3 and aging [158]. Actually, previous studies have already shown that vitamin D3 was able to improve the aged-associated impaired spermatogenesis of D-gal-induced-aging rats [143] and to alleviate H_2_O_2_-induced DNA damage [144]. In addition, it was found that the sterol compounds isolated from the mussel *Mytilidae*, including cholesterol (CHOL), brassicasterol, crinosterol and 24-methylenecholesterol, could all expand the lifespan of yeast, exhibiting apparent anti-aging activity [145].

#### 2.4.3. Fungal Sterols

Fungi are rich in bioactive compounds, including sterols. Fungal sterols are widely distributed in edible mushrooms [146,159], Chinese cordyceps [147] and yeast [160]. The main type of fungal sterols are ergosterols, which can be used for the synthesis of vitamin D and steroid hormones in animals. Ergosterols are also widely applied in pharmaceuticals, advanced biofuels and perfume ingredients [159].

Several studies have shown that fungal sterols have anti-aging activity. It was shown that ergosterol derivatives, ganodermasides A and B, isolated from the spores of the glossy Ganoderma *Ganoderma lucidum*, extended the replicative lifespan of the yeast *Saccharomyces cerevisiae* [146]. Cerevisterol, another sterol isolated from Chinese cordyceps, not only improved the survival rate of H9C2 cells under hypoxia but also exhibited anti-aging effects in mice [147].

### 2.5. Terpenoids

**Figure 2:**
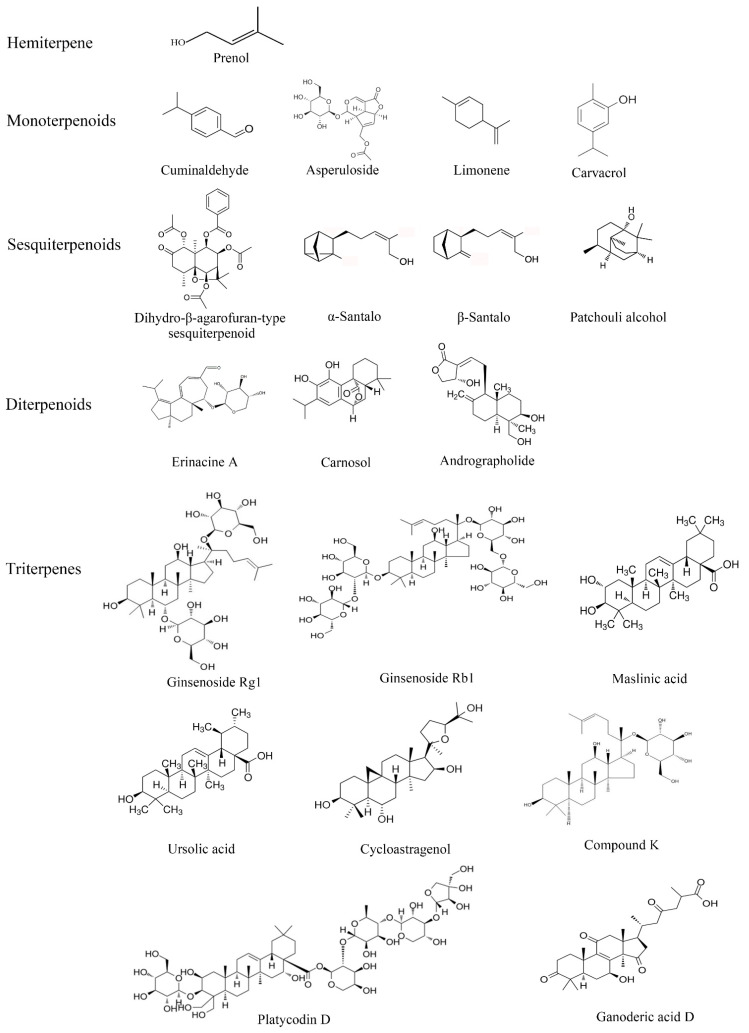
The chemical structures of different types of terpenoids.

Terpenoids, composed of five carbon isoprene units, are a large class of natural products that includes more than 40,000 structures widely used in the flavor, fragrance, chemical and pharmaceutical industries [161]. Based on their structures, terpenoids are divided into eight subclasses: hemiterpenoids, monoterpenoids, sesquiterpenoids, diterpenoids, sesterterpenoids, triterpenoids, tetraterpenoids and polyterpenoids [162]. Previous studies have shown that terpenoids have multiple biological effects, including anti-cancer, antioxidant, anti-inflammatory, antibacterial, neuroprotective, anti-diabetic and anti-aging activities [163,164,165,166,167,168,169]. Now, we will describe the anti-aging activity of several representative terpenoids from natural food sources. Table 5 lists the representative terpenoids with possible anti-aging mechanisms, and Figure 2 shows the chemical structures of the different types of terpenoids.

#### 2.5.1. Hemiterpenoids

The hemiterpene compound 3,3-dimethylallyl alcohol (prenol) is an unsaturated prenyl alcohol and one of the simplest members of the terpenoid family. It is present in citrus fruits like lemon, orange and pomelo, where it serves as an aroma constituent and acts as a key precursor of several biological molecules [198]. Prenol has recently been reported to possess anti-aging potential. Prenol supplementation was found to extend the lifespan of wild-type *C. elegans* up to 22.8% compared to control worms [170]. Moreover, suspended amyloid-β-induced paralysis and reduced α-synuclein aggregation were observed in prenol-treated worms. This lifespan-prolonging property of prenol appeared to be correlated with ameliorated physiological parameters and increased stress (heat and oxidative) tolerance in the nematodes. Both in-silico and gene-specific mutant studies revealed that the longevity transcription factors DAF-16, HSF-1, and SKN-1 were involved in the improved lifespan and health span of prenol-treated worms.

#### 2.5.2. Monoterpenoids

Cuminaldehyde, an aromatic monoterpenoid volatile compound, is an aromatic oily aldehyde found in essential oils obtained from the cumin *Cuminum cyminum* [171]. Cuminaldehyde was shown to be able to upregulate the expression of *Bdnf*, *Icam* and *ApoE* but downregulate the expression of *IL-6* in the brains of aged rats, suggesting that cuminaldehyde has a neuroprotective effect [171]. Asperuloside is the main bioactive compound of iridoid, belonging to monoterpenoid extracted from the Chinese herb Du Zhong *Eucommia ulmoides* male flower. It was reported that asperuloside could delay the muscle aging of *C. elegans* through a DAF-16-mediated improvement in mitochondrial dysfunction [172]. Limonene is a naturally occurring cyclic monoterpene, available in a variety of fruits and vegetables, such as citrus and conifer plants. Limonene was able to protect HaCaT cells (human skin keratinocytes) against UVB-induced photodamage and photo-aging through the activation of the Nrf2-dependent antioxidant defense system [173]. Carvacrol (CAR) is a phenolic monoterpenoid present in the essential oils of the oregano *Origanum vulgare*, the thyme *Thymus vulgaris*, the pepperwort *Lepidium flavum* and the wild bergamot *Citrus aurantium bergamia* [199]. CAR was found to decrease the content of MDA and the relative mRNA expression of *p53*, *p21* and *B-cell lymphoma-2 associated X protein (Bax)*; to increase the activity of total antioxidant capacity (TAC) and GPX; and to upregulate the level of glutathione S-transferase (GST) in the brains of D-gal-induced aged rats. CAR was thus considered a promising natural protective compound capable of delaying aging and maintaining health [174].

#### 2.5.3. Sesquiterpenoids

Dihydro-β-agarofuran-type sesquiterpenoids are the primary secondary metabolites in the seeds of the medicinal plant genus *Celastrus* [175,200]. Twelve dihydro-β-agarofuran-type sesquiterpenoids have been purified from the seeds of *Celastrus virens*, and compounds 1, 2, 5, 6, 8, and 9 (at 50 μM) were found to extend the mean survival time of *C. elegans*. It was further revealed that the extension of lifespan mediated by compounds 1, 6, 8, and 9 was dependent on the transcription factors skn-1 and hsf-1 [175]. Both α- and β-santalol are the most abundant sesquiterpenoids found in the sandalwood *Santalum paniculatum*. Treatment with α- and β-santalol was shown to retard aging, improve health span, and inhibit the aggregation of toxic amyloid-β (Aβ_1−42_) and polyglutamine repeats (Q35, Q40, and HtnQ150) in a *C. elegans* models for Alzheimer’s and Huntington’s disease, respectively. Moreover, α- and β-santalol selectively regulated the transcription factors SKN-1/Nrf2 and EOR-1/promyelocytic leukemia zinc finger (PLZF) through the receptor tyrosine kinase/Ras GTPase/MAP kinase (RTK/Ras/MAPK)-dependent signaling axis capable of triggering the expression of several antioxidant genes and protein aggregation inhibitory genes, such as *gst-4*, *gcs-1*, *gst-10*, *gsr-1*, *hsp-4*, and *skr-5*, which contribute to extending longevity and helping minimize age-induced protein oxidation and aggregation [176]. Patchouli alcohol (PA), a kind of sesquiterpene extracted from the patchouli *Pogostemon cablin*, was also shown to produce an anti-aging effect. PA could inhibit D-gal-induced oxidative stress and ameliorate the quality of aging cartilage via the activation of the Nrf2/HO-1 pathway in mice [177].

#### 2.5.4. Diterpenoids

Erinacine A (EA) is a diterpenoid derivative extracted from the mycelium of the mushroom *Hericium erinaceus* (HEM). EA has been reported to reverse spatial learning disabilities in aging mice. It was demonstrated that both HEM- and EA-treated mice had shorter mean daily escape latencies than control group mice. HEM- and EA-treated mice also showed a decrease in the expression of *TNF-α* and *IL-1β*. Moreover, HEM and EA could reduce body weight, abdominal fat, plasma glucose, serum and liver total cholesterol and liver triacylglycerol. Thus, HEM and EA may be potential health-promoting supplements for minimizing the progression of aging and obesity-induced neurodegeneration by decreasing metabolic abnormalities and neuroinflammatory cytokines and increasing neurogenesis factors [178]. Carnosol, a phenolic diterpene, is one of the main constituents of the aromatic herb *Rosmarinus*. Carnosol was able to increase the health span of *C. elegans*. It effectively decreased ROS accumulation under normal or oxidative stress conditions, significantly increased the activity of CAT, SOD and GPX, and reduced MDA content. Moreover, carnosol-mediated longevity required the upregulated expression of *sod-3*, *sod-5*, *hsf-1*, *hsp-16.1*, and *hsp-16.2*, and was dependent on *hsf-1* gene. This suggests that carnosol can be used as a potential dietary supplement to slow down aging [179]. Andrographolide (ANDRO) is a diterpenoid lactone, which is the main active constituent of the Chinese traditional herb Chuan Xin Lian *Andrographis paniculata*. ANDRO administration could increase the recognition memory and preference for novel experiences, restore social recognition and long-term memory, and enhance basal synaptic activity and long-term potentiation (LTP) in aged female degus (a long-lived rodent) *Octodon degus*. Moreover, ANDRO increased the level of N-methyl D-aspartate receptor subtype 2B (NR2B) in both adult and aged degus, and the level of post-synaptic density protein-95 (PSD95) and Homer1 (homer scaffolding protein 1) in aged degus. Thus, the long-term administration of ANDRO is able to improve age-dependent behavioral and synaptic dysfunction impairment in long-lived female degus [180].

#### 2.5.5. Triterpenes

Compound K (CK) is a secondary ginsenoside biotransformed from major ginsenosides of the herb *Panax ginseng*, which is more bioavailable and soluble than its parent ginsenosides. Ginsenoside CK was found to improve the memory function in scopolamine-injured mice by regulating Aβ aggregation and promoting the transduction of the Nrf2/Keap1 signaling pathway, thereby resulting in a reduction in oxidative damage to neurons and the inhibition of neuronal apoptosis [181]. Similarly, CK treatment attenuated chronic cerebral hypoperfusion (CCH)-induced Aβ_1–42_ deposition and ameliorated cognition impairment in vascular-dementia rats by upregulating the expression of both pSer9-glycogen synthase kinase 3β (pSer9-GSK3β) and insulin-degrading enzyme (IDE), which are responsible for the production and clearance of Aβ_1–42_ [182]. This suggests that CK may serve as a potential agent for the prevention and treatment of aging-induced Alzheimer’s disease. In addition, CK supplementation could diminish the production of COX-2 and metalloproteinase-1 (MMP-1) in NIH3T3 cells treated with UV-B and modulate the expression of type I collagen [183]. Rg1, another kind of ginsenoside, also possesses anti-aging activity [184,201]. It was found that Rg1 could exert anti-aging functions by suppressing mitochondrial pathway-mediated apoptosis and activating the sirtuin 3 (SIRT3)/SOD2 pathway in Sca-1⁺ HSC/HPC cells in a D-gal-induced-aging rat model [184]. Moreover, 20-O-β-D-glucopyranosyl20(S)-protopanaxadiol (20GPPD), the primary bioactive metabolite of ginsenoside Rb1, enhanced the production of hyaluronic acid by acting as an upstream modulator of ERK and Akt activity mediated by Src kinase in human keratinocytes [186].

Ganoderic acid D (GA-D) is a triterpenoid compound produced by the Ganoderma *G. lucidum*. It was shown that GA-D treatment inhibited the activity of SA-β-Gal, decreased the ROS level and the expression of P21 and P16 proteins, relieved the cell-cycle arrest, and enhanced the telomerase activity in senescent human amniotic mesenchymal stem cells (hAMSCs). Furthermore, GA-D was able to alleviate the senescence of hAMSCs through the activation of the CaM/CaMKII/Nrf2 (Ca^2+^ calmodulin/CaM-dependent protein kinase II/nuclear erythroid 2-related factor 2) signal pathway [187,188]. Maslinic acid (MA) is a triterpene derivative obtained from the olive tree *Olea europaea*. MA showed antioxidant activity and was capable of reducing the endogenous plasma lipoperoxide levels and susceptibility to lipid peroxidation in rats, thus offering some advantages in the anti-aging process [189]. Ursolic acid (UA) is a natural triterpenoid compound extensively present in apple peels. UA was found to promote skeletal muscle rejuvenation by enhancing SIRT1 expression and satellite cell proliferation [190,202]. Moreover, UA treatment also reversed the aging of the liver by enhancing SIRT1 and SIRT6 levels and promoting the production of PGC-1β (peroxisome proliferator-activated receptor γ coactivator 1) and Klotho [191]. Cycloastragenol (CA) is a natural triterpenoid saponin compound obtained from the milkvetch root *Astragali radix*. Both CA and its new derivatives could significantly extend the lifespan of *C. elegans* [192].

### 2.6. Vitamins

Vitamins are a structurally dissimilar group of organic compounds that share the common features of being essential for normal cellular functions, growth and development. Most vitamins cannot be produced in human body, and they must be obtained through dietary intake. Vitamins are divided into two groups: water-soluble vitamins and fat-soluble vitamins. The former are soluble in water and polar solvents and mainly consist of B vitamins and vitamin C, and the latter are insoluble in water but soluble in fat and organic solvents and comprise vitamins A, D, E and K [203]. Previous studies have shown that vitamins have multiple biological effects, including antioxidant, anti-aging, anti-inflammatory, anti-nociceptive and anti-cancer properties [204,205]. Here, we describe the anti-aging activities of water-soluble vitamins and fat-soluble vitamins from natural food sources and their possible anti-aging mechanisms (Table 6).

#### 2.6.1. Water-Soluble Vitamins

B vitamins include vitamins B1 (thiamin), B2 (riboflavin), B3 (nicotinic acid/niacinamide), B5 (pantothenic acid), B6 (pyridoxine), B7 (biotin), B9 (folate) and B12 (cobalamin), which are grouped together due to their water solubility, though they are a diverse group of molecules with varied metabolic functions related to energy production, protein metabolism and hemopoiesis [215]. Chemically, riboflavin (RF) is 7, 8-dimethyl-10-ribityl-isoalloxazine and consists of a flavin isoalloxazine ring bound to a sugar side chain, ribitol [216]. RF, or vitamin B2, is heat-stable. Cooking does not lower levels of RF; however, exposure to light could destroy it. RF can be found in a wide variety of foods and from natural sources, especially in milk and organ meats—mostly in calf liver, egg, fish, nuts, certain fruits and legumes, wild rice, mushrooms, dark green leafy vegetables, yeast, beer, cheese and dietary products [217,218]. Previous studies have demonstrated that RF has anti-aging effects. For example, the supplementation of RF was shown to significantly extend the lifetime and strengthen the reproduction of fruit flies by enhancing the activity of antioxidant enzymes [206]. PVN, a topical liquid formula of polydeoxyribonucleotide (PDRN), vitamin C, and niacinamide (a kind of vitamin B3 derivant), was shown to be capable of resisting UV-B-induced skin aging. PVN treatment increased the expression of Nrf2/HO-1 and the activity of SOD and decreased the activities of nicotinamide adenine dinucleotide phosphate hydrogen oxidase and tyrosinase, as well as the expression of tumor protein p53 and microphthalmia-associated transcription factor, in UV-B-radiated animal skin. PVN also decreases melanin content in the skin. Moreover, PVN treatment decreased the expression of the nuclear factor kappa-light-chain enhancer of activated B cells and MMP2/3/9 but increased the expression of the collagen type I α1 chain [207].

Vitamin C, or ascorbic acid, is an essential dietary nutrient and one of the naturally occurring antioxidants in nature [219,220]. It has been shown that vitamin C is capable of repairing DNA, alleviating oxidative stress, and modulating the activity of telomeres in the aging process, thereby eventually leading to longevity [221]. Vitamin C was generally used as a supplement in cosmetic and dermatologic products for anti-skin-aging purposes [208,222,223,224]. For instance, a split-face, randomized controlled trial indicated that vitamins C and E improved skin color, elasticity, radiance, smoothness, scaliness, and wrinkles of fifty female volunteers aged 30–65 years [225]. Additionally, it was reported that vitamin C delayed liver aging by reducing lipid deposition [209]. Vitamin C administration also decreased the expression of fatty acid synthase (FAS), acetyl-CoA carboxylase (ACC), and ATP-citrate lyase (ACL), and reduced the contents of total cholesterol ratio (TC), triglycerides (TGs) and non-esterified fatty acids (NEFAs) through the regulation of GSK-3β/mTOR signaling in the liver of zebrafish treated with LiCl [209].

#### 2.6.2. Fat-Soluble Vitamins

Retinol is the bioavailable form of vitamin A and has been reported to possess the ability to reduce skin discoloration, stimulate collagen production, reduce rhytids, and improve acne and uneven skin texture, thus exhibiting anti-skin-aging activity [226,227]. Recently, retinol has also been shown to exert anti-skin-aging activity by decreasing glycan metabolism, enhancing protein synthesis/folding, and being involved in cytoskeletal rearrangement [228].

Vitamin E includes eight structurally related lipid-soluble compounds with potent antioxidant properties, consisting of four tocopherols and four tocotrienols: α (alpha), β (beta), γ (gamma) and δ (delta) [229]. α-Tocopherol is the most abundant and bioavailable form of vitamin E in humans and rodents [230,231] and serves as a strong ROS scavenger that protects polyunsaturated fatty acids in cellular membranes and lipoproteins from peroxidation [232]. In a DNA repair-deficient mutant mouse (*Xpg*^−/−^), vitamin E supplementation exhibited an obvious neuroprotective effect. Vitamin E resulted in a delayed onset of age-related body weight decline and the appearance of tremors and considerably prevented DNA damage-induced liver abnormalities such as changes in polyploidy. Additionally, the intake of vitamin E reduced the number of p53-positive cells throughout the brain, indicative of a lower number of cells dying due to DNA damage accumulated over time [210]. A previous study also reported that vitamin E, or velvet antler polypeptide, could significantly improve the learning and cognitive abilities of D-gal-induced-aging mice, increase the activities of SOD, GPX, and CAT in the serum, and decrease the MDA content. Intestinal microecological analysis showed that vitamin E, or velvet antler polypeptide, markedly increased the abundance of the beneficial bacterial genus *Lactobacillus*. Metabolism analysis showed that vitamin E, or velvet antler polypeptide, promoted fatty acid metabolism by activating peroxisome proliferator-activated receptor α (PPARα) and upregulating the expression of the downstream enzymes carnitinepalmitoyl transferase-1 A and acyl-CoA oxidase 1 while downregulating that of apolipoprotein E4 (APOE4). Thus, vitamin E, or velvet antler polypeptide, is able to exert anti-aging activity through the modulation of the gut microbiota and the regulation of the PPARα/APOE4 pathway [211]. Similarly, dietary quercetin and vitamin E were also shown to improve intestinal function in aged breeder hens by protecting intestinal structure and integrity [212]. Quercetin and vitamin E exerted synergistic effects on intestinal morphology by promoting villi height and crypt depth, mitigating the intestinal inflammatory damage of the aged hens, decreasing the concentration of serum D-lactate and diamine oxidase, and increasing the levels of secretory immunoglobulin A and *Mucin-2* mRNA. Furthermore, the expression of intestinal tight junction protein genes including *occludin*, *ZO1*, and *claudin-1* was increased by quercetin and vitamin E. Quercetin and vitamin E could also lower the expression of the pro-inflammatory genes (*TNF-α*, *IL-6*, and *IL-1β*) and increase the expression of anti-inflammatory genes (*IL-10* and *IL-4*). Moreover, quercetin and vitamin E protected the small intestinal tract from oxidative damage by increasing the levels of SOD, GPX, and CAT and the expression of *SOD1* and *GPX-2*, and decreased MDA levels in the intestine [212]. Vitamin E was also reported to be able to prevent age-related neuronal disorders by suppressing oxidative stress and enhancing antioxidant enzyme activity [233,234], protecting the integrity of the skin barrier, thus possessing anti-skin-aging activity [235,236,237].

Vitamin K is another kind of fat-soluble vitamin with a common chemical structure: a 2-methyl-1,4-naphthoquinone ring and a variable aliphatic side chain. The variable aliphatic chain differentiates two isoforms: vitamin K1, or phylloquinone, and vitamin K2, usually designated as menaquinone. Vitamin K is abundant in dark green leafy vegetables, several fruits (e.g., dried prunes, kiwifruit, avocado, blueberries, blackberries, grapes), some nuts (pine nuts, cashews, pistachios), cheeses, eggs, and meats [238]. Previous studies demonstrated that vitamin K is a vital cofactor in activating several proteins that act against aging and age-related diseases [239,240]. Thus, vitamin K status is closely associated with aging and age-related diseases [241,242,243]. Vitamin K was initially discovered as a co-factor for γ-glutamyl carboxylase (GGCX), which could add a carboxyl group to the gamma-position carbon of glutamate residues in the substrate proteins [244,245]. A recent study demonstrated that vitamin K could alleviate aging-related musculoskeletal disorders such as osteoporosis, osteoarthritis, and sarcopenia by enhancing GGCX activity [246]. Meanwhile, other modes of vitamin K actions, such as the regulation of transcription by activating steroid and xenobiotic receptor (SXR), physical association to 17β-hydroxysteroid dehydrogenase type 4 (17β-HSD4), covalent modification of Bcl-2 antagonist killer 1 (Bak) and modulation of protein kinase A (PKA) activity, were also reported [246]. Vitamin K could improve aging and aging-related diseases through its antioxidant properties. Treatment with vitamin K was shown to decrease ROS and lipid peroxidation levels, increase the glutathione (GSH) level and the activities of both GPX and alkaline phosphatase (ALP), and promote DNA proliferation in human osteoblasts cultured in the presence of hydroxyapatite-based biomaterials that cause oxidative stress [213]. A 4-week randomized controlled trial in middle-aged and older individuals indicated that dietary intake of vitamin K-rich green leafy vegetables decreased the fracture risk of individuals by substantially reducing serum total osteoblasts, osteocalcin (OC) and undercarboxylated OC, which suggests the increased entry of OC into the bone matrix, where it may improve the material properties of bones [247]. An increase in the intake of dietary vitamin K was associated with better cognitive function scores in an older adult Mediterranean population with high cardiovascular risk [248] and in adults with chronic kidney disease [249]. Recently, vitamin K2 was reported to extend the lifespan of *C. elegans* and improve the resistance to pathogenic infection, heat stress and H_2_O_2_-induced inner oxidative stress [214]. Interestingly, the roles of vitamin K2 on aging and stress resistance were shown to be dependent on enhanced fat metabolism but not due to its antioxidant effects, suggesting that vitamin K2 could enhance fat degradation and digestion to improve survival, supporting the effectiveness of vitamin K2-based medical treatments [214].

## 3. Anti-Aging Mechanisms of Compounds from Food Sources

We have provided a brief introduction to compounds from natural food sources, including polysaccharides, polyphenols, carotenoids, sterols, terpenoids and vitamins above. They have been shown to display anti-aging effects in different organism models, including yeast, nematode, fruit flies, fish, hens and mice. Now, we will discuss their modes of anti-aging action. Currently, the anti-aging mechanisms of the compounds are mainly found to be associated with several modes of action, such as improved antioxidant capacity, regulation of age-related gene expression, improved immune function, regulation of apoptosis, intestinal flora regulation, regulation of autophagy and suppression of cellular senescence.

### 3.1. Suppression of Oxidative Stress

All animals produce ROS during normal physiological activity, especially in the course of oxidative phosphorylation. At the same time, ROS-scavenging systems including the antioxidant enzymes CAT, SOD and GPX also run in parallel in the body. However, the balance between the ROS and ROS-scavenging systems is becoming increasingly difficult to maintain with increased age, resulting in an excess of ROS. Excess ROS can cause the peroxidation of lipids, the oxidation of proteins, and DNA and mitochondria damage, thereby impairing cellular integrity and functionality, ultimately leading to the deteriorated health status of an organism and accelerating aging [250]. The Keap1/Nrf2/AREs (Kelch-like ECH-associated protein 1/nuclear factor erythroid 2-related factor 2/antioxidant-response elements) signaling pathway is a defensive transduction system for animals to regulate oxidative stress. Keap1 is an inhibitor of Nrf2. Normally, Nrf2 is bound to Keap1 and remains inactive in cytoplasm [251]. When stimulated by internal and external free radicals or chemicals, the conformation of Keap1-Nrf2 changes and Nrf2 dissociates from Keap1 and translocates into the nucleus and interacts with the AREs, eventually activating a series of antioxidant genes such as heme oxygenase-1 (HO-1), nicotinamide adenine dinucleotide phosphate (NADPH), GST, SOD, and GPX [252,253].

It is known that polysaccharides, polyphenols, carotenoids, sterols, terpenoids and vitamins can all have anti-aging effects through the suppression of oxidative stress. Polysaccharides, including LBP, CPs (polysaccharides of the dangshen *Codonopsis pilosula*), SCPs (sulfated polysaccharides of *C. pilosula*) and SFPs (polysaccharides of the hijiki *Sargassum fusiforme*), were all able to exert anti-aging activity through the activation of the Keap1/Nrf2/ARE pathway, which enhances antioxidant system activity, ultimately resulting in the scavenging of ROS, a reduction in DNA damage, and the protection of lipids and proteins from oxidation [34,40,49,50]. Polyphenols such as quercetin, rutin, anthocyanin, resveratrol, flavonoid and phenolic acids also exhibited antioxidant activity by regulating the production and activity of endogenous antioxidant enzymes SOD, GPX, GSH, NADP(H) quinone oxidoreductase 1 (NQO1), GST, and HO-1, which involves the Keap1/Nrf2/AREs-mediated pathway [79,80,254,255]. Carotenoids like lutein were shown to protect cells from H_2_O_2_-induced senescence by promoting the expression of antioxidant effectors such as NADPH quinone dehydrogenase 1, HO-1 and sirtuins [125]. Interestingly, astaxanthin (ATX) exerted anti-aging effects through the regulation of both antioxidative activity and anti-immunosenescence activity, which are mediated by Keap1/Nrf2 and NF-κB pathways, respectively [118]. The sterol vitamin D3 was found to improve age-associated spermatogenesis impairment through the regulation of antioxidant system activity via enhancing SOD, GSH and CAT activity [143], and treatment with vitamin K and D3 decreased the ROS and lipid peroxidation levels and increased the glutathione (GSH) level and the activity of GPX in the presence of hydroxyapatite-based biomaterials that cause oxidative stress [213]. Notably, we found that DG extended both the mean and maximum lifespans of the aged male fish *N. guentheri* through the enhancement of the activity of antioxidant enzymes, including CAT, SOD and GPX; the promotion of the proteolytic activity of the ubiquitin–proteasome pathway; and the suppression of the phosphatidylinositol 3-kinase/protein kinase/molecular target of the rapamycin (PI3K/AKT/mTOR) signaling pathway [138]. The monoterpenoid limonene and terpenoid platycodin D both suppressed intracellular ROS generation by increasing the production of endogenous antioxidant enzymes [173,194], which are associated with enhanced Nrf2. Similarly, the terpenoid ginsenoside Re (GRe) attenuated increased NADPH oxidase (NOX) activity and ROS levels, increased GPX activity, and improved cognitive dysfunction in *Klotho*-deficiency-induced-aging mice via the upregulation of Nrf2/GPX signaling [195].

### 3.2. Regulation of Age-Related Genes and Pathways

Quite a few genes, including those coding for Sirtuin1 (SIRT1), cellular tumor antigen p53 (p53), cyclin-dependent kinase inhibitor 1 (p21) and the 66-kDa isoform of ShcA (p66Shc), and several pathways, such as the IIS pathway and the rat sarcoma/protein kinase A (Ras/PKA) pathway, have been identified as related to aging and longevity. Mutations and/or changes in the expression of the genes and pathways can significantly impact the lifespan of organisms.

SIRT1 is a member of the sirtuins (SIRTs) family, which plays a protective role in the development of many age-related diseases, including cardiovascular disease, neurodegeneration and cancer [256,257]. Previous studies have shown that the expression of SIRT1 leads to an increase in the DNA stability and survival rate of animals [258]. SIRT1 has been found to regulate many biological processes, including cellular senescence, apoptosis, glucose homeostasis, aging and longevity. The protein p53, a short-lived tumor suppressor, is normally ubiquitinated rapidly by mouse double minute 2 protein (MDM2), a p53-specific E3 ubiquitin ligase, and subsequently targeted for degradation by the ubiquitin–proteasome system. In contrast, in the presence of DNA damage, p53 is activated by post-translational modifications, which inhibit the interaction of p53 with MDM2 and lead to the stabilization and accumulation of p53. As p53 not only triggers apoptosis but also induces DNA damage, the expression of the p53 gene is thus related to both cell senescence and cell-cycle arrest, thereby exerting an influence on aging [259]. The cell-cycle inhibitor p21 (CDKN1A) is involved in many important cellular processes, including apoptosis and DNA replication. CDKN1A is also a cell-cycle regulatory protein capable of inhibiting the activities of various cell-cycle-dependent kinases, ultimately resulting in cell-cycle arrest [260]. Polysaccharides have been reported to affect the aging process by interfering with the cell cycle via the regulation of the expression of both SIRT1 and p53 as well as the CDKN1A genes. For example, the polysaccharide extracted from the root of Yulangsan *Millettia pulchra Kurz* (YLSP) was able to reduce the expression of p53 and p21 genes in D-gal-induced mice, which is responsible for the delay of liver and brain senescence [41]. *Tremella fuciformis* polysaccharides (TFPs) could attenuate hydrogen peroxide (H_2_O_2_)-induced cell oxidative stress and apoptosis through the upregulation of the SIRT1 pathway [60], and LBP alleviated cell apoptosis and aging through the p53-mediated pathway [36]. Similarly, β-carotene reduced aging rates in tissues and organs in vivo and appeared to inhibit aging caused by antioxidative stress by regulating KAT7-P15 signaling [112], and the polyphenol resveratrol protected the impairment of learning and memory ability and suppressed neuronal apoptosis in aged rats induced by sevoflurane and nitrous oxide through upregulating the expression of Sirt1 [87]. Interestingly, the water-soluble vitamin B3 derivant niacinamide also exhibited a protective effect against UV-light-induced DNA damage and aging in epidermal melanocytes by increasing the components of various signaling pathways, such as Sirt1 and Nrf2 [261].

p66Shc, a growth factor adapter protein, has been shown to affect ROS production by reducing ROS scavenging, by increasing membrane oxidase activity, or through mitochondrial respiratory chain leakage. It can also suppress the expression of antioxidant genes by inhibiting Forkhead box O 3a (FoxO3a) activity [262,263,264]. Our previous study demonstrated that β-1,3-glucans administration increased the activities of antioxidant enzymes, including CAT, GPX, and SOD, by decreasing the level of p66Shc. This resulted in the reduction of ROS levels, which in turn slows down protein oxidation, lipid peroxidation, LF, and SA-β-Gal development, eventually extending the lifespan of aging fish [58]. Similarly, polyphenol caffeic acid (CA) and dihydrocaffeic acid (DHCA) promoted longevity and increased stress resistance in *C. elegans* by activating the DAF-16/FoxO transcription factor and modulating the expression of stress-related genes [76], and the sterol β-sitosterol attenuated aging sarcopenia via regulating FoxO1-dependent signaling in a mouse model [141]. Notably, the terpenoid ginsenoside Rg1 exerted anti-aging effects by suppressing mitochondrial pathway-mediated apoptosis and the SIRT3/SOD2 pathway in Sca-1⁺ HSC/HPC cells of an aging rat model [184].

The IIS pathway is closely associated with the aging and longevity of animals. Normally, IIS is active and is mainly involved in the phosphorylation of DAF-2, AGE-1 and other kinases, thus affecting the activity of DAF-16 [265], a transcription factor mediating the aging rate of animals like *C. elegans* [266]. Any condition that causes internal stress to block the IIS pathway will induce DAF-16 to enter the nucleus; in the nucleus, DAF-16 binds to the promoters and regulates the transcription of its target genes, which are predominantly stress-resistant genes and longevity-promoting genes such as *sod-3*, *skn-1*, *sir-2.1* and *hsp-16.2*, thereby delaying senescence and prolonging the lifespan of animals [267,268]. Several studies demonstrated that polysaccharides prolonged the lifespan of *C. elegans* and *D. melanogaster* through the regulation of the IIS/FoxO signal pathway [32,39,45,47,269].

The Ras/PKA pathway regulates several aspects of cellular physiology, including growth and responses to glucose and stress, through the phosphorylation of different transcription factors that are also mediated by other signaling pathways [270]. The hexose transporter 7 gene, *hxt7*, can be used as a reporter of Ras/PKA activity, and the expression of *hxt7* is repressed when the Ras/PKA pathway is active [271]. In addition, the Ras/PKA pathway also mediates redox response through the downregulation of the expression of cytoplasmic catalase T (CTT1) and mitochondrial SOD2. It has been demonstrated that both GFGs and HEGs exert anti-aging and neuroprotective effects through the inhibition of the Ras/PKA pathway [52]. Polysaccharides can also affect the lifespan and aging of animals by regulating other lifespan-related signaling pathways. For example, LBP was shown to exert its anti-aging activity by activating MAPK and inhibiting the TOR/S6K (target of rapamycin/ribosomal s6 protein kinase) signaling pathway [31]. Similarly, tart cherry extract (TCE, the main component of anthocyanins) significantly increased the lifespan of *C. elegans* through the alteration of the AMPK/AMP-activated protein kinase (AAK-2) and DAF-16/IIS pathways [82], and the terpenoids prenol and dihydro-β-agarofuran-type sesquiterpenoid both extended the lifespan of *C. elegans* through the downstream transcription factors of the IIS pathway, HSF-1 and SKN-1 [170,175].

### 3.3. Immune Modulation

Aging impairs immunity, resulting in overall dysfunctions. This has been termed immunosenescence, manifested by age-related defects in both humoral and cell-mediated immune responses [272]. Meanwhile, senescent cells usually produce excessive inflammatory cytokines such as IL-1β, interleukin-1 (IL1), TNF-α, and chemokines like C-X-C motif ligand 1 (CXCL1), leading to serious inflammatory responses [273]. Many studies have demonstrated that polysaccharides exert anti-aging activity by enhancing immunity function while suppressing inflammatory response [41,274]. LBP was shown to alleviate the neuroinflammation of estrogen deficiency-induced-aging mice through the downregulation of IL-6, IL-1β and TNF-α levels by inhibiting the toll-like receptor 4/nuclear factor-kappa B (TLR4/NF-κB) signaling pathway [33], and RGBPs were found to attenuate skin aging caused by UV and atopic dermatitis by increasing antioxidant activity, inhibiting activating protein-1 (AP-1) transactivation, and reducing chemokine MDC/CCL22 and TARC/CCL17 levels [43]. Our previous study demonstrated that β-1,3-glucans exerted anti-aging activity by regulating the immune system via enhancing levels of T helper cells (CD4^+^) and delayed-type hypersensitivity (DTH) response, a T cell-mediated immune response, as well as promoting the production of total anti-keyhole limpet hemocyanin (KLH) immunoglobulin G (IgG), anti-KLH IgG1 and anti-KLH IgG2a levels in aged mice without disturbing immune homeostasis [59].

Polyphenols, sterols, carotenoids, terpenoids and vitamins also exert an anti-aging effect though improving inflammatory response. The polyphenol resveratrol administration delayed the aging of the fish *N. guentheri*, reversed the increase in SA-β-Gal activity with aging, downregulated the levels of senescence-associated secretory phenotype (SASP)-associated proinflammatory cytokines IL-8 and TNF-α, and upregulated the expression of anti-inflammatory cytokine IL-10 [88]. In addition, resveratrol exerted anti-aging effects by regulating both humoral and cell-mediated immune responses. It demonstrated that the dietary intake of resveratrol induced a significant increase in CD4^+^ cells in middle-aged and aged Wistar male rats, increased the DTH response in aged rats, and remarkably promoted the production of total anti- KLH IgG, anti-KLH IgG1, and anti-KLH IgG2a in aged rats without disturbing immune homeostasis [89]. The carotenoid trans-lycopene (from tomato juice) alleviated the risk of aging-associated cardiovascular disease by reducing the concentration of important inflammatory molecules related to atherosclerosis [115], and lycopene showed protective effects in the learning and memory functional deficits of Aβ-induced Alzheimer’s disease rats by reducing the expression of toll-like receptor 4 (TLR4) and NF-κB p65 and the levels of TNF-α, IL-1β, and IL-6β in serum [116]. The sterol β-sitosterol showed an anti-aging effect by preventing TNF-α-induced gonadotropin-releasing hormone (GnRH) decline through the inhibition of NF-κB activation [142]. Krill oil, rich in ATX, exerted its anti-aging and rejuvenation effects via the enhancement of the antioxidant system and the suppression of the NF-κB signaling pathway [122]. The terpenoid Rg1 ameliorated aging-induced liver fibrosis in SAMP8 mice through the inhibition of NADPH oxidase 4/NACHT, LRR, and PYD domain-containing protein 3 (NOX4/NLRP3) inflammasome activation [196]. Similarly, quercetin and vitamin E improved intestinal function in aged breeder hens by protecting the intestinal structure and integrity through the suppression of inflammation and the enhancement of antioxidant activity [212].

### 3.4. Regulation of Apoptosis

Apoptosis plays an important role in the maintenance of homeostasis when intracellular or extracellular damage occurs. On one hand, non-functional and damaged cells can be scavenged by activated apoptosis, and on the other hand, apoptosis dysregulation, along with aging, contributes to age-related pathologies. Thus, apoptosis is involved in aging and age-related diseases [18,275]. Polyphenols have been shown to affect aging and age-related diseases by regulating apoptosis. For example, quercetin alleviated H_2_O_2_-induced vascular smooth muscle cell (VSMC) senescence by regulating apoptosis through P53-P21 and P16 pathways [78], and resveratrol improved the pancreas aging of senescence-accelerated mice (SAM) by modulating the inflammatory, oxidative and apoptotic status related to aging [90].

Carotenoids, sterols and terpenoids also showed anti-aging effects through the regulation of apoptosis. The carotenoid astaxanthin was found to enhance the longevity of *Saccharomyces cerevisiae* by decreasing oxidative stress and apoptosis [120]. Astaxanthin alleviated glutamate excitotoxicity-related neuronal loss associated with Alzheimer’s disease by regulating oxidative stress and apoptosis through the Akt/glycogen synthase kinase-3b (GSK-3β) signaling pathway [121]. The sterol vitamin D3 delayed the testicular senescence of a D-gal-induced-aging rat model by regulating proliferation and apoptosis [143]. The terpenoid ginsenoside Rg1 ameliorated spatial learning and memory deficits in D-galactose and AlCl3-induced-aging mice by restoring fibroblast growth factor 2-protein kinase B (FGF2-Akt) and the brain-derived neurotrophic factor-tropomyosin-related kinase B (BDNF-TrkB) signaling axis to inhibit apoptosis [197]. Similarly, ginsenoside GRb1 retarded the aging process in natural-aged C57BL/6J mice by regulating the cell cycle and apoptotic pathway, which were associated with the alleviation of metabolic disorders [185]. Moreover, the monoterpenoid carvacrol (CAR) delayed the aging rates of D-gal-induced-aging rats by decreasing the expression of P53, P21 and Bax, leading to the inhibition of apoptosis [174].

### 3.5. Intestinal Flora Regulation

The gut microbiota of animals shows a wide inter-individual variation, but its within-individual variation is relatively stable over time. With the advance of chronological age, the overall richness of gut microbiota in animals decreases, while a certain group of bacteria associated with frailty increases. Studies using model animals have shown that age-related gut dysbacteriosis, or dysbiosis for short, is contributable to unhealthy aging and reduced longevity [276]. It has been reported that the risk of cognitive decline and neurodegenerative diseases caused by aging can be reduced through the regulation of the gut microbiota and metabolites. For example, an increase in the abundance of *Faecalibacterium prausnitzii*, *Eubacterium* and *Roseburia*, induced by the Mediterranean diet, was positively associated with several markers of improved cognitive function [277,278]. Polysaccharides can be entirely fermented by the intestinal microbiota and increase the number of probiotic bacteria, ultimately benefiting healthy aging. WMPs were reported to significantly improve the locomotor activity and spatial and recognition memory of aging mice by both alleviating oxidative stress and decreasing pro-inflammatory cytokine levels, as well as increasing the short-chain fatty acid (SCFA) level and abundance of the beneficial bacteria *Bacteroides* and *Parabacteroides* [55]. FLAPs and FHSPs were demonstrated to prolong the lifespan of *C. elegans* by improving the antioxidant defense system and enriching specific gut bacteria (*Romboutsia* and *Weissella*) and metabolites [51]. Similarly, GRPs were found to extend lifespan and alleviate the aging of *C. elegans* by increasing antioxidant activity, altering the composition of gut flora, and inducing the preferential synthesis of beneficial fatty acids [38]. Interestingly, vitamin E also exerted anti-aging activity through the modulation of the gut microbiota and regulation of the PPARα/APOE4 pathway [211].

### 3.6. Autophagy Regulation

Macroautophagy, often simply referred to as autophagy, exists widely in eukaryotic cells and is closely associated with many physiological and pathological processes. In the protein degradation machinery, autophagy is primarily involved in the degradation of long-lived proteins and, as a major cellular self-degradation process, helps remove damaged or excessive proteins and organelles. During autophagy, autophagic vacuoles are fused with lysosomes to generate autolysosomes, and the cellular debris and damaged proteins are then degraded in autolysosomes. Mitophagy, a form of selective autophagy, is a crucial mechanism for controlling mitochondrial quality through the removal of dysfunctional or damaged mitochondria. Both autophagy and mitophagy play a dual role in aging and aging-associated diseases. When they are weakened, they cause aging and aging-related diseases; when they are too strong, they induce the autophagy death of normal cells [279,280].

Bioactive compounds from natural food sources can work as essential autophagy modulators and thus be proficiently used in the control of aging and aging-related diseases, in which autophagy plays a crucial role. In 6-hydroxydopamine (6-HODA)-induced PC12 cells (Parkinson’s disease model cells), the expression of the key regulator of autophagy, LC3-II, was decreased, and this could be reversed by treatment with APS (*astragalus* polysaccharide) through the PI3K/AKT/mTOR pathway [281]. APS also provided a strong protective effect against hepatocyte senescence and pathological damage in aged mouse livers by reducing ROS levels, inhibiting apoptosis and pyroptosis, and promoting mitophagy via the AMPK/mTOR pathway [282]. It was also found that the *Echinacea purpurea* polysaccharide could improve oxidative stress-induced liver injury by promoting phosphatase and tensin homolog (PTEN)-induced putative kinase 1 (PINK1)/E3 ubiquitin ligase (Parkin)-dependent mitophagy [282]. The protein levels of LC3-II and Beclin were abundantly expressed in a Methyl-4-phenyl-1,2,3,6-tetrahydropyridine (MPTP)-induced Parkinson’s disease (PD) mice model, suggesting the overactivation of autophagy, but they were significantly downregulated by *Lycium barbarum* polysaccharide (LBP) treatment [283]. Similarly, pretreatment with LBP also reduced PM2.5-induced mitophagy and mitochondrial damage [35]. The *Ganoderma lucidum* polysaccharide was also shown to ameliorate MPTP-induced Parkinsonism and protect dopaminergic neurons from oxidative stress via regulating mitochondrial function, autophagy, and apoptosis [284].

Interestingly, green tea polyphenols also protected against early vascular senescence in young high-fat-diet rats by ameliorating oxidative injury and promoting autophagy, which was partially regulated by the SIRT3 pathway [285]. In *C. elegans* models of PD, chlorogenic acid (CGA), a polyphenolic substance derived from various medicinal plants, showed anti-PD effects through autophagy induction via increasing the expression of autophagy-related genes, including *unc-51*, *bec-1*, *vps-34*, and *lgg-1* [286]. Oroxin B (OB), a flavonoid isolated from traditional Chinese herbal medicine, alleviated aging-related osteoarthritis (OA) through anti-inflammation, the inhibition of the PI3K/AKT/mTOR signaling pathway and the enhancement of autophagy [287]. Resveratrol was also shown to suppress age-related sarcopenia and cardiomyocyte hypertrophy through the restoration of autophagy in aged mice via the activation of SIRT1 [288]. Similarly, cucurbitacin B (CuB), containing cerebrosides, phenols, sesquiterpenoid, triterpenoids, and sterols isolated from natural products, was reported to prolong both the replicative lifespan and chronological lifespan in yeast by regulating autophagy, ROS, antioxidative ability, and aging-related gene expression [289].

### 3.7. Suppression of Cellular Senescence

Cellular senescence refers to an intrinsic aging process in which cells undergo irreversible growth arrest due to their inherent finite lifespan or exposure to stress [11,290]. The number of senescent cells increases exponentially with age in various tissues and organs, playing a critical role in the development and pathogenesis of age-related diseases. Cellular senescence generally acts as an inherent anti-cancer mechanism because senescent cells lose the ability to proliferate and perform apoptosis in response to DNA damage [291]. However, senescent cells remain highly metabolically active and exhibit a hypersecretory state known as the senescence-associated secretory phenotype (SASP). The SASP is mainly composed of proinflammatory cytokines, chemokines, proteases, lipids, growth factors and insoluble proteins or extracellular matrix (ECM) components, which are simultaneously often used as markers to identify cellular senescence accurately [292]. The primary role of the SASP is to recruit immune cells to the site of damage and clear the senescent cells through inflammatory mechanisms. As a result, the SASP molecules work transiently in a beneficial manner to perform immune surveillance, promote tissue repair and homeostasis, and suppress tumorigenesis [292]. A recent study showed that the efficient induction of senescence in DNA-damaged cells avoids a potential sub-population of damaged cells, eventually leading to the extension of lifespan [293]. However, an accumulation of senescent cells with age, or chronic senescence, is accompanied by a high rate of secretion of SASP factors, which frequently exceeds the immune system’s clearance capacity [294]. The improper disposal of senescent cells or overactivation of the SASP can result in detrimental effects and increase the risk of disease and mortality [295].

Accumulating data indicated that bioactive compounds from natural food sources exert anti-aging effects by mediating cellular senescence. Fucoidan, a marine-sulfated polysaccharide, was shown to reverse the *p*-cresol-induced cellular senescence of mesenchymal stem cells (MSCs) via the regulation of SMP30 and p21 (the senescence-associated proteins) and increase proliferation through the regulation of cell-cycle-associated proteins cyclin-dependent kinase 2 (CDK2), cyclin-dependent kinase 4 (CDK4), cyclin D1, and cyclin E, which are activated through the focal adhesion kinase (FAK)-Akt-class A basic helix-loop-helix protein 38 (TWIST) axis [296]. In the D-gal pre-administrated rats, treatment with the *Angelica sinensis* polysaccharide (ASP) rescued the senescence of hematopoietic cells and bone marrow stromal cells (BMSCs), reserved myeloid progenitor cells, attenuated stromal oxidative load, ameliorated stem cell factor CXCL12 and granulocyte-macrophage colony-stimulating factor (GM-CSF) production, and increased connexin-43 (Cx43) expression. A further study indicated that ASP ameliorated D-gal-induced senescence by inhibiting the secretion of IL-1, IL-6, TNF-α, and the chemokine RANTES, enhancing Cx43-mediated intercellular communication, and improving runt-related transcription factor 2 (Runx2) expression, while decreasing peroxisome proliferator-activated receptor gamma (PPARγ) expression in BMSCs [297]. The senolytic compounds, dasatinib and quercetin, were found to prevent an age-dependent progression of disc degeneration in mice by preserving cell viability, phenotype, and matrix content via the regulation of the levels of cellular senescence markers p16^INK4a^ and p19^ARF^; SASP molecules IL-6 and MMP13 [298]; and the natural plant polyphenols, curcumin and quercetin. This ameliorated skin cellular aging induced by UV irradiation and H_2_O_2_ stimulation through activating the TGF-β and Nrf2 signaling pathways, enhancing collagen production and antioxidant enzyme expression, and decreasing the expression of MMP-1, MMP-3, COX-2, TNF-α and IL-1 [299]. Cocoa polyphenols extract (CPE) was reported to attenuate senescent phenotypes in an H_2_O_2_-induced cellular senescence model by downregulating SA-β-Gal expression, promoting cell proliferation, and decreasing oxidative DNA damage and mitochondrial dysfunction by inhibiting mitochondrial reactive oxygen species (mtROS) generation through the mediation of SIRT1, SIRT3, FoxO3, and P53 [300]. β-Cryptoxanthin (BCX), a type of carotenoid, attenuated H_2_O_2_-induced oxidative stress and cellular senescence in HK-2 cells by promoting Nrf2 nuclear translocation [301]. Vitamin D3 attenuated doxorubicin-induced senescence and cell-cycle arrest in human aortic endothelial cells through the upregulation of IL-10 and FoxO3a expression via the fine modulation of p-AMPKα/SIRT1/FoxO3a complex activity [302]. Betulinic acid (BA), a kind of pentacyclic triterpenoid, is abundant in fruits such as *Zizyphus* sp., *Dillenia* sp., and *Azanza* sp. Treatment with BA promoted cellular proliferation and alleviated the cellular senescence of normal human dermal fibroblasts (NHDFs) via the inhibition of the type I interferon signaling pathway [303].

### 3.8. Other Anti-Aging Mechanisms

Food-derived bioactive compounds have also been found to exert anti-aging activity through other modes of action in addition to the mechanisms mentioned above. Telomeres are specialized structures found at the ends of linear chromosomes, containing a tandemly repeated sequence (TTAGGG) and associated protective proteins. Telomeres shorten with each cell division, and the main function of telomeres is to protect chromosome ends from fusion and degradation in proliferating cells [304,305,306]. Telomerase, a unique reverse transcriptase in cells, is able to add repetitive telomeric sequences de novo onto telomeric ends, thus leading to telomere restoration [307]. Numerous studies have proved that telomeres continually shorten with the aging process, which is also a risk factor for many age-related diseases [307]. Hence, telomerase activation in vivo has significant potential for delaying aging and alleviating aging-associated diseases [308,309,310,311]. For example, the terpenoid cycloastragenol (CA) and its biotransformation products showed anti-aging effects by improving telomerase activation [193,312,313]. Similarly, the polysaccharide extracted from *Angelica sinensis* polysaccharide (ASP) and *Astragalus membranaceus* polysaccharide (AMP) were shown to exert anti-aging activity by improving telomerase activity and regulating p53-p21 and p16-pRb pathways [44]. A recent study, which used cross-sectional data from the National Health and Nutrition Examination Surveys (NHANES) database from 1999 to 2002, also indicated that vitamin C was positively correlated with telomere length, with greater dietary vitamin C intake being associated with longer telomeres in a total of 7094 participants [314].

Mitochondria are essential not only for obtaining ATP from glucose and fatty acids but also in amino acid metabolism, pyridine synthesis, phospholipid modification and calcium regulation [19]. It is well known that mitochondrial dysfunction and decreased mitochondrial biogenesis are hallmarks of aging and play a role in speeding aging [315,316]. Polyphenols have been found to be able to delay aging through regulation of mitochondrial function and biogenesis. Resveratrol significantly extended the lifespan of mice fed a high-calorie diet by improving mitochondrial function [317]. Similarly, the polysaccharides from ginseng alleviated the aging of *C. elegans* by inducing the preferential synthesis of beneficial fatty acids [38], and the polysaccharides from the brown alga *Sargassum fusiforme* (SFPs) protected liver tissue in old male mice by improving energy metabolism [48]. Additionally, the polysaccharide from Chinese wolfberry *Lycium barbarum* reduced PM2.5-induced cell death and ameliorated cell morphology through the regulation of apoptosis and mitochondrial autophagy [35].

## 4. Conclusions and Perspectives

Human beings are entering an aging society globally. With the constant increase in the social pension burden, the discovery and development of safe and effective anti-aging approaches to prolong healthy lifespan are receiving increasing attention. Numerous studies have shown that both nutritional and pharmacological interventions can extend lifespan in a variety of model organisms, including yeast, nematodes, fruit flies, fish, mice and rats. In this review, we summarized the bioactive compounds from natural food sources that have the potential to prolong lifespan and prevent aging-associated diseases. These compounds include polysaccharides, polyphenols, carotenoids, sterols, terpenoids and vitamins, which are abundant in edible plants, algae, animals and fungi. The compounds have dual use in both food and medicine and show great potential for maintaining elderly health and prolonging healthy lifespan. However, there are still many issues that need to be studied and solved before they can be truly applied. The primary issues are as follows:

1. Most of the compounds are mixtures and therefore need further purification in order to determine the exact molecule that exhibits the bioactive activity.
2. A compound often has multiple modes of action. Therefore, for specific compounds, their modes of action require in-depth research.
3. Lifespan extension is a direct and key indicator for evaluating the anti-aging efficiency of bioactive compounds. It is relatively easy to detect the lifespan of animals with short life cycles such as nematodes and fruit flies, but it is difficult to do so for human beings as they have much longer lifespans. Therefore, we need to search for and identify suitable and intuitive anti-aging indicators to examine the life-extension effects of the compounds on humans.
4. Previous anti-aging experiments are often limited to research on the short-term effects of the compounds. Therefore, long-term and large-scale clinical trials are urgently needed to investigate the anti-aging effects of compounds.

## Figures and Tables

**Table 1:** Anti-aging effects and modes of action of edible polysaccharides.

Subclasses of Polysaccharides	Representative Components	Models	Main Indicators of Aging	Modes of Action	Reference
Plant polysaccharides	Chinese wolfberry *Lycium barbarum* polysaccharide (LBP)	*D. melanogaster*	Lifespan; MDA content; CAT and SOD activity; The relative mRNA expression of aging-related genes (*mapk*, *tor* and *s6k*) and longevity genes (*hep*, *mth* and *rpn11*).	LBP exerts anti-aging activity by regulation of age-related genes and signaling pathway.	[31]
	LBP	*C. elegans*	In-vitro study: 2,2-diphenyl-1-(2,4,6-trinitrophenyl) hydrazyl (DPPH), hydroxyl, superoxide anion radical scavenging activity assay. In-vivo study: Lifespan; Oxidative-stress resistance; Heat-stress resistance; The relative mRNA levels of *hsp-16.2*, *sod-3*, *daf-16* and *daf-2*.	LBP prolongs the lifespan of *C. elegans* by upregulating the expression of *daf-16*, *sod-3* and *hsp-16.2*.	[32]
	LBP	Aging mice induced by estrogen deficiency	Learning and memory impairment assay; Levels of myeloid differentiation factor 88, NF-κB, TNF-α, IL-6 and IL-1β associated with TLR4/NF-κB signaling pathway.	LBP alleviates estrogen deficiency-induced cognitive impairments by downregulating the factors associated with TLR4/NF-κB signaling pathway.	[33]
	LBP	UVB-irradiation-treated HaCaT cells	Cell viability; ROS level; DNA damage; Levels of p-p38, p38, mmp-9, caspase-3 and Nrf2; The relative mRNA expression of Nrf2 target genes (*AKR1C2*, *APOE*, *GCLC*, *GCLM*, *HBEGF*, *HO-1* and *NQO1*); SOD activity.	LBP protects against UVB irradiation-induced photodamage partially through activation of Nrf2/ARE pathway and suppression of UVB-induced p38 MAPK pathway.	[34]
	LBP	PM2.5-treated HaCaT cells	Cell viability and apoptosis assay; ROS level; MDA content; SOD activity; Mitochondrial damage; Autophagosome level.	LBP protects skin cells from PM2.5-induced cytotoxicity by regulation of oxidative stress-ER stress-autophagy-apoptosis signaling axis.	[35]
	LBP	Zebrafish embryos	SA-β-gal activity assay; Survival rate assay; The relative mRNA expression of *p53*, *p21*, *Bax*, *Mdm2* and *TERT*.	The effects of LBP on cell apoptosis and aging might be mediated by the p53-mediated pathway.	[36]
	Ginseng *Chuanminshen violaceum* polysaccharides (CVPs)	D-Gal-induced-aging mice	In-vitro study: DPPH, hydroxyl, and superoxide anion radicals scavenging activity assay. In-vivo study: Body weight and organ indexes assay; T-SOD, Mn-SOD, Cu/Zn-SOD and CAT activity assay; MDA content; The relative mRNA expression of *Cu/Zn-SOD*, *Mn-SOD*, *CAT*, *GPx*, *thioredoxin 1* (*Trx1*), and *thioredoxin 2* (*Trx2*).	CVPs show anti-aging effect by increasing antioxidant activity.	[37]
	Ginsenoside residue polysaccharides (GRPs)	*C. elegans*	Lifespan assay; LF and ROS level; SOD activity; Microbiomic and transcriptomic analysis.	GRPs prolong lifespan and alleviate aging of *C. elegans* without affecting locomotive behaviors by increasing antioxidant activity, altering composition of gut flora and inducing preferential synthesis of beneficial fatty acids.	[38]
	Sweet tea *Rubus suavissmus* polysaccharides (STP-60a)	*C. elegans* and its mutant strains	Lifespan assay; LF and MDA content; The survival rate under heat stress and oxidative stress assay; Pharyngeal pump rate; The activities of SOD, CAT and GPX; ROS level; The relative mRNA expression of *daf-16*, *skn-1*, *hsf-1*, and their target genes.	STP-60a activates the autophagy system of nematodes through insulin and mitochondrial signaling pathways, enhances the antioxidant capacity, and ultimately promotes the longevity of *C. elegans*.	[39]
	Dangshen *Codonopsis pilosula* polysaccharides (CPs); Sulfated *Codonopsis pilosula* polysaccharides (SCPs)	H_2_O_2_-treated RAW 264.7 cells; Mice with acute oxidative injury	Cell viability and apoptosis assay; Biochemical index assay; SOD, CAT and GPX activity; MDA and ROS level; The relative mRNA expression of *Keap1* and *Nrf2*.	Anti-aging activities of CP and SCP are mediated by activating Keap1/Nrf2 signaling pathway to enhance activity of antioxidant system.	[40]
	Yulangsan *Millettia pulchra Kurz* polysaccharide (YLSP)	D-Gal-induced-aging mice	General appearance, body weight, and organ index assay; The activities of SOD, CAT, T-AOC and GPX; The level of MDA, IL-2, IL-6 and AGEs; The content of p53 and p21.	YLSP may have a protective effect suppressing the aging process by enhancing antioxidant activity and immunity, as well as modulating aging-related gene expression.	[41]
	*Akebia trifoliata* fruit polysaccharides (ATFPs)	*C. elegans*	In-vitro study: 2,2′-azino-bis (3-ethylbenzothiazoline-6-sulfonate) (ABTS) and hydroxyl radical-scavenging assay. In-vivo study: Lifespan; SOD, CAT, GPX activity; MDA and ROS level; LF content; mRNA levels of *sir-2.1*, *daf-16*, and *daf-12*.	ATFPs prolong lifespan of *C. elegans* through improving activity of antioxidant enzymes, that are regulated by *sir-2.1*, *daf-16*, and *daf-12* in insulin/IGF signaling pathway.	[42]
	Red ginseng by-product polysaccharides (RGBPs)	HaCaT cells; SD rats; Male NC/Nga mice with atopic dermatitis-like symptoms and skin lesions	ABTS radical scavenging activity; Toxicity test; Cell viability; MMP-1, MMP-2 and AP-1 content; Typical Th2 chemokine (MDC/CCL22 and TARC/CCL17) level.	RGBPs alleviate skin aging caused by sUV and atopic dermatitis through increasing antioxidant activity, inhibiting AP-1 transactivation, and reducing MDC/CCL22 and TARC/CCL17 level inducted by TNF-α/IFN-γ.	[43]
	*Angelica sinensis* polysaccharide (ASP); *Astragalus membranaceus* polysaccharide (AMP)	H_2_O_2_-treated human fetal lung fibroblast WI-38 cells	Cell viability assay; SA-β-gal activity assay; Telomerase activity assay; Cell-cycle analysis; The content of p53 and p16^INK4^.	ASP and AMP exert anti-aging activity through improving telomerase activity and regulating p53-p21 and p16-pRb pathways.	[44]
Algal polysaccharides	Kelp *Laminaria japonica* polysaccharide (LJP)	*C. elegans*	In-vitro study: DPPH, ABTS and hydroxyl radical-scavenging assay. In-vivo study: Lifespan assay; MDA, ROS, and LF level; T-SOD activity; The relative mRNA expression of *Sod-3*, *Glp-1*, *Skn-1*, *Daf-2*, *Daf-16* and *Age-1*; Metabolic profiling.	LJP prolongs lifespan of *C. elegans* and shows anti-aging activity by resisting oxidative stress and promoting alanine, aspartate, and glutamate metabolism, TCA cycle, butanoate metabolism and FoxO signaling pathway.	[45]
	Red alga *Pyropia haitanensis* polysaccharide, porphyrin	AD mice	In-vitro study: DPPH-radical scavenging assay. In-vivo study: Learning and memory assay; Activity of AChE and ChAT.	Porphyrin ameliorates learning and memory impairment of AD mice by increasing cerebral acetylcholine content in cortical and hippocampal tissue.	[46]
	Asparagus *Gracilaria lemaneiformis* polysaccharides (GPs)	*C. elegans*	Lifespan assay; Reproduction; Pharyngeal pumping; Thermotolerance; Oxidative stress; Polyglutamic acid content; DAF-16 level.	Anti-aging activity of GPs is not dependent upon calorie restriction but on insulin pathway DAF-16.	[47]
	Hijiki *Sargassum fusiforme* polysaccharides (SFPs)	Old male ICR mice	Proteomics study.	SFPs promote longevity of mice, which is intensively related to amino acid metabolism, antioxidation and energy metabolism.	[48]
	SFPs	HL-7702 cell line; D-Gal-induced-aging mice; Male chronic aging mice	In-vitro study: DPPH and hydroxyl radical-scavenging assay; Cells viability assay. In-vivo study: Ub-Nrf-2, Nrf-2 and p21 level; The relative mRNA expression of *Nrf2*, *Keap1*, *Nqo1*, *Mn-SOD*, *CuZn-SOD*, *GCLM*, *GCLC* and *GPx1*.	SFPs decelerate aging process by enhancing Nrf2-dependent cytoprotection, especially antioxidant defense.	[49]
	SFPs	Young, middle-aged and aged male ICR mice	The relative mRNA expression of *Nrf2*, *SOD-2*, *CAT*, *NQO1* and *HO-1*; Small intestine microbiota assay.	SFPs ameliorate declined cytoprotective capacity of small intestine in aging process of mice by upregulating the Nrf2/ARE signaling pathway and partially rejuvenating the overall status of small intestine microbiota.	[50]
Fungal polysaccharides	Bolete *Lanmaoa asiatica* polysaccharides (FLAPs); Yellow mushroom *Hohenbuehelia serotina* polysaccharides (FHSPs)	*C. elegans*	Lifespan assay; ROS, LF and MDA level; SOD and CAT activity; Specific gut bacteria and metabolites assay.	FLAPs and FHSPs prolong lifespan of *C. elegans* by improving the antioxidant system and enriching specific gut bacteria and metabolites without reproductive toxicity.	[51]
	Maitake *Grifola frondosa* β-glucans (GFGs); Monkey head mushroom *Hericium erinaceus* β-glucans (HEGs)	Yeast cells; *D. melanogaster*	Lifespan assay; ROS level; The relative mRNA levels of *RAS2*, *HXT7*, *CTT1*, and *SOD2*; α-Synuclein toxicity and its aggregation.	β-Glucans prolong lifespan through inhibiting the Ras/PKA pathway, reducing the toxicity of α-synuclein, and suppressing its aggregation.	[52]
	*Grifola frondosa* intracellular zinc polysaccharides (IZPs)	D-Gal-induced-aging mice	In-vitro study: Hydroxyl radical, DPPH radical, superoxide radical, hydrogen peroxide-scavenging assay; Antibacterial activity. In-vivo study: T-AOC and SOD activity; MDA content; Histopathology analysis.	IZPs show anti-aging effect by increasing antioxidant and antibacterial activities of mice.	[53]
	Golden mushroom *Flammulina velutipes* sulfated polysaccharides (SFPs)	D-Gal-induced-aging mice	In-vitro study: Hydroxyl radical, DPPH radical, superoxide radical-scavenging effect assay. In-vivo study: Hematological parameters and serum biochemical indexes assay; SOD, GPX, CAT and T-AOC activity; MDA and LPO content; AchE and nitric oxide synthase (NOS) activity; Histopathology analysis.	SFPs show protective abilities against aging by increasing antioxidant activities, improving inflammatory response and ameliorating the anile condition of mice without disturbing general condition and physiology.	[54]
	White button mushroom *Agaricus bisporus* polysaccharides (WMPs)	D-Gal-induced-aging mice	Animal behavioral tests; SOD and GPX activity; MDA content; TNF-α, IL-1β and IL-6 level; Gut microbiota assay; Short-chain fatty acid (SCFA) level.	WMPs improve locomotor activity and the spatial and recognition memory of aging mice by alleviating oxidative stress, decreasing pro-inflammatory cytokine levels, and increasing the SCFA level and abundance of beneficial bacteria.	[55]
	*Agaricus bisporus* acidic-extractable polysaccharides (AcAPs)	D-Gal-induced-aging mice	Serum biochemical indexes assay; SOD, GPX, CAT activity; MDA content; Histopathology analysis.	AcAPs show anti-aging effects by increasing antioxidant activity and alleviating oxidative stress.	[56]
	*Agaricus bisporus* enzyme-assisted polysaccharides (EnAPs)	D-Gal-induced-aging mice	In-vitro study: Reducing power assay; hydroxyl radical and DPPH radical-scavenging effect assay; Fe^2+^-chelating rates. In-vivo study: SOD, GPX, CAT activity; MDA content; Histopathology analysis.	EnAPs show anti-aging effects by increasing antioxidant activity, improving organ function and remitting lipid metabolism.	[57]
	β-1,3-Glucans	Aged *N. guentheri*	Lifespan assay; LF content; SA-β-Gal activity; ROS, protein oxidation and lipid peroxidation level; CAT, SOD and GPX activity; The level of p66Shc.	β-1,3-Glucans show anti-aging effects by increasing antioxidant activity.	[58]
	β-1,3-Glucans	Young, middle-aged and aged mice	DTH response assay; Hematologic index and biochemical parameter assay; The levels of KLH-specific IgG, IgG1 and IgG2a; Cellular composition of splenocytes and proliferation of splenocytes assay.	β-1,3-Glucans exert anti-aging effects by enhancing the adaptive immune responses of aged mice without disturbing their general condition and physiology.	[59]
	*Tremella fuciformis* polysaccharides (TFPs)	H_2_O_2_-treated human dermal fibroblasts-neonatal (HDF-n)	Cell viability and apoptosis assay; ROS level; Levels of p16, p21, p53 and SIRT-1.	TFPs attenuate H_2_O_2_-induced cell oxidative stress and apoptosis through the upregulation of the SIRT1 pathway.	[60]

**Table 2:** Anti-aging effects and modes of action of polyphenols.

Subclasses of Polyphenols	Representative Components	Models	Main Indicators of Aging	Modes of Action	Reference
Phenolic acids	Chlorogenic acid (CGA) extracted from coffee and tea	*C. elegans*	Lifespan assay; Thermotolerance and stress resistance assay; The relative mRNA expression of FoxO transcription factors *daf-16*, *hsf-1*, *skn-1* and *hif-1*.	CGA extends lifespan of *C. elegans* via DAF-16 in insulin/IGF-1 signaling pathway.	[74]
	p-Coumaric acid	*C. elegans*	Lifespan assay; Stress resistance assay; ROS level; The relative mRNA expression of *daf-16*, *skn-1*, *sod-3*, *osr-1*, *osm-7*, *osm-11* and *ama-1*.	p-Coumaric acid increases oxidative resistance of *C. elegans* by regulating *skn-1*, an ortholog of Nrf2.	[75]
	Caffeic acid (CA); Dihydrocaffeic acid (DHCA)	*C. elegans*	Lifespan and stress assay; The relative mRNA expression of the genes *daf-16*, *daf-18*, *skn-1*, *ctl-1*, *hsp-16.2*, *hsf-1*, *sod-3*, and *sir-2.1*.	CA and DHCA promote longevity and increase stress resistance in *C. elegans* by activating the DAF-16/FoxO transcription factor and modulating the expression of stress-related genes.	[76]
Flavonoids	Quercetin	*C. elegans*	Lifespan assay; Evaluation of motility; Stress resistance assay; ROS level; The relative mRNA expression of *daf-2*, *age-1*, *daf-16*, *nsy-1*, *sek-1*, *pmk-1*, *skn-1* and *hsf-1*.	Quercetin induces heat-stress tolerance in *C*. *elegans* by modulating HSF-1 expression and/or activity.	[77]
	Quercetin	H_2_O_2_-induced senescence vascular smooth muscle cell (VSMC)	Cell viability assay; SA-β-gal activity; Apoptosis assay; The content of Bax and Bcl-2.	Quercetin alleviates H_2_O_2_-induced VSMC senescence by regulating apoptosis through the P53-P21 and P16 pathways.	[78]
	Quercetin; Rutin	AD model mice	GSH/GSSG ratio; MDA content; CAT, SOD and GPX activity; BACE1 activity; The relative mRNA expression of *cat*, *sod*, *gpx*, *APP*, *BACE1*, *ADAM10*, *caspase-3*, *caspase-6*, *IL-1β*, *TNF-α* and *IFN-γ*.	Quercetin and rutin both alleviate the aging phenotype of AD mice by augmenting intracellular redox homeostasis in the brain.	[79]
	Anthocyanins from purple wheat	*C. elegans* and its mutant strains	In-vitro study: DPPH free radical scavenging assay. In-vivo study: Lifespan assay; The relative mRNA expression of *hsp-16.2*; ROS level; DAF-16 location.	Anthocyanins extend the lifespan of *C. elegans* mainly by regulating the DAF-16/FoxO signaling pathway.	[80]
	Anthocyanins from honeysuckle *Lonicera pallasii*	*D. melanogaster*	Lifespan assay; Stress resistance, locomotor activity and intestinal integrity assay; The relative mRNA expression of *eEF1α2*, *RpL32*, *Hsp27*, *Hsp68*, *Hsp83*, *Keap1*, *NRF*, *Sod1*, *HIF1*, *Clk* and *Per*; Radical scavenging and antioxidant activity assay.	Honeysuckle anthocyanins prolong lifespan and improve the health span of *D. melanogaster* through enhancing antioxidant activities.	[81]
	Tart cherry extract (TCE)	*C. elegans* and its mutant strains	Lifespan assay; The relative mRNA expression of *daf-16*, *daf-2*, *daf-18*, *aak-2*, *akt-1*, *lin-14*, *skn-1*, *ucp-4*, *sod-2/3*; Mitochondrial respiration analysis.	TCE confers health span benefits to *C. elegans* through enhanced mitochondrial function and reduced oxidative stress, mainly via the DAF-16 pathway.	[82]
Stilbenes	Resveratrol	*D. melanogaster*	Longevity and fecundity assay; Locomotor activity; SOD and CAT activity assay.	Resveratrol prolongs the lifespan of both male and female adult fruit flies by scavenging ROS and neuroprotection through increasing antioxidant enzyme activity.	[83]
	Resveratrol	Aged *N. guentheri*	Lifespan assay; Cognitive ability and locomotor activity assay; Neurofibrillary degeneration assay; SA-β-gal activity; LF content.	Resveratrol prolongs the lifespan of aged fish by retarding neurodegeneration and aging-related histological markers.	[84]
	Resveratrol	Aged *N. guentheri*	SA-β-gal activity; LF content; Histological evaluation of ovarian development; PCNA, SIRT1 and GRP78 content; The relative mRNA expression of *NRF2*, *NF-κB*, *IL-1β*, *TNF-α*, *IL-8*, *GRP78* and *CHOP*.	Resveratrol activates SIRT1/NRF2 to reduce inflammation and ER stress so as to delay ovarian aging in *N. guentheri*.	[85]
	Resveratrol	HtrA2 knockout mice	Motor phenotype assay; Survival assay; The relative mRNA expression of *chop*, *atf4*, *p53*, *noxa*, *bcl2*, *bax*, *dr5*; NeuN staining assay.	Resveratrol alleviates neural aging by attenuating apoptosis at the level of Bax.	[86]
	Resveratrol	18-month-old SD rats anaesthetized by sevoflurane and nitrous oxide	Learning and memory assay; The content of SIRT1, Poly ADP-ribose polymerases-1 (PARP-1), cleaved caspase-3 and Bax.	Resveratrol improves learning and memory ability and inhibits neuronal apoptosis by increasing the expression of SIRT1 in aged rats after general anesthesia.	[87]
	Resveratrol	6-, 9-, 12-month-old *N. guentheri*	SA-β-gal activity assay; The relative mRNA level of *IL-8*, *IL-10*, *SIRT1*, *NF-κB*; The content of TNFα, SIRT1, RelA/p65, p-IκBα, IκBα and LGR5.	Resveratrol delays the aging of the annual fish *N. guentheri* by inhibiting SASP through the SIRT1/NF-κB signaling pathway.	[88]
	Resveratrol	Young, middle-aged and aged rats	DTH response assay; Hematologic index and biochemical parameter assay; The levels of KLH-specific IgG, IgG1 and IgG2a; Cellular composition of splenocytes and proliferation of splenocytes assay.	Resveratrol exerts an anti-aging effect by enhancing the adaptive immune responses of aged rats without disturbing their general condition and physiology.	[89]
	Resveratrol	Senescence-accelerated mice (SAM)	The relative mRNA expression of *TNF-α*, *IL-1β*, *IL-10*, *Bcl-2-associated death promoter* (*BAD*), *Bcl-2-associated X protein* (*BAX*), *B-cell lymphoma 2* (*Bcl-2*), *X-linked inhibitor of apoptosis protein* (*XIAP*), *SIRT1*, *FoxO1* and *FoxO3A*; The content of TNF-α, IL-1β, IL-10, NF-κB p65, NF-κB p50–105, NF-κB p52–100, inhibitor of NF-κB (IκB)-β and -α, BAD, BAX, and Bcl-2; The activity of GPX, glutathione reductase (GR), and glutathione transferase (GST); Plasma glucose and insulin assay.	Resveratrol improves the pancreas aging of SAM by modulating the inflammatory, oxidative and apoptotic status related to aging.	[90]
Lignans	Sesamin from sesame seed	β-Amyloid-induced-aging *C. elegans* model	Aβ-induced paralysis assay; The relative mRNA expression of Aβ transgene; β-Amyloid content; Chemotaxis behavior assay; Lifespan assay.	Sesamin prolongs the lifespan of an β-amyloid-induced-aging *C. elegans* model by reducing Aβ toxicity.	[91]
	Sesamin	*C. elegans* Bristol strain N2 and its mutant strains	Lifespan assay; Bacterial infection assay; Locomotory scoring assay; Stress resistance assay; LF content; Protein oxidant assay; Brood size assay.	Sesamin enhances the host defense of *C. elegans* and increases average lifespan via the activation of both skn-1 (encoding a component of the p38 MAPK pathway) and daf-16 (encoding a component of the IGF-1 pathway).	[92]
Tannins	Tannic acid (TA)	*C. elegans* strain N2 and its mutant strains	Lifespan assay; Stress resistance; Nematode length and reproduction assay; Pharynx pumping rate.	TA influences the aging process by regulating *sek-1*.	[93]
	ellagic acid (EA)	*C. elegans* strain N2 and its mutant strains	Lifespan assay; Stress resistance; Antimicrobial capacity assay; Length and reproduction assay; Pharynx pumping rate; Triglyceride assay.	EA influences the aging process by regulating antimicrobial effects and hormetic action.	[94]
	Oenothein B (OEB)	Wild-type *C. elegans* strain and its mutant strains	Lifespan assay; Reproduction assay; Pharynx pumping rate; Locomotion and thermotolerance assay; ROS level; SOD content; Age pigment content.	OEB might modulate multiple genetic pathways involved in insulin/IGF-1 signaling (IIS) via *age-1* and *daf-16*, the dietary restriction (DR) pathway via *eat-2* and *sir-2.1*, and the mitochondrial electron transport chain via *isp-1* to promote healthy lifespan.	[95]
	Pentagalloyl glucose (PGG)	Wild-type *C. elegans* strain and its mutant strains	Lifespan assay; Reproduction assay; Pharynx pumping rate; Locomotion and thermotolerance assay; ROS level; SOD content; Age pigment content.	PGG and its metabolites promote healthy lifespan by regulating the IIS and DR pathway and the mitochondrial electron transport chain.	[96]

**Table 3:** Anti-aging effects and modes of action of carotenoids.

Subclasses of Carotenoids	Representative Components	Models	Main Indicators of Aging	Modes of Action	Reference
Carotenes	β-Carotene	Aged MSCs induced by H_2_O_2_; Aged mice	In-vitro study: Content of p16 and p21; DNA damage and cell proliferation assay; Levels of IL-1 β, IL-6 and TNF-α; Levels of ROS and MDA; SOD activity; Level of KAT7 and P15. In-vivo study: Psychology and physiology behaviors of aged mice; Inflammation and tissue fibrosis levels.	β-Carotene inhibits aging by regulating the KAT7-P15 signaling axis.	[112]
	Lycopene	10-month-old adult mice	Hematology and clinical biochemistry indexes assay; MDA content; CAT, SOD and GPX activity.	Lycopene alleviates aging by increasing antioxidant enzyme activity.	[113]
	Lycopene	D-Gal-induced and naturally aging hens (*Gallus domesticus*)	Morphological and ultrastructure assay; Somatic cell proliferation assay; Levels of Bax, Bcl-xL, PCNA, CDK2 and CCND1; GSH, ROS and MDA contents; T-SOD, CAT, GPX and T-AOC activity; Levels of Nrf2, p-Nrf2, HO-1 and NQO1.	Lycopene ameliorates the aging of hen ovaries by inhibiting oxidant stress and apoptosis via the activation of the Nrf2/HO-1 pathway.	[114]
	trans-Lycopene from tomato juice	28 patients (mean age 69.7 ± 3.1 years; mean BMI 31.5 ± 3.6 kg/m^2^) at high cardiovascular risk	Plasmatic carotenoids, ICAM-1, VCAM-1, and C-reactive protein (CRP) levels assay.	Trans-lycopene may attenuate the risk of cardiovascular disease by reducing the concentration of important inflammatory molecules related to atherosclerosis.	[115]
	Lycopene	Aβ_1–42_-induced AD rats	Behavioral parameters assay; Serum levels of TNF-α, IL-1β and IL-6β; The mRNA expressions of *TLR4*, *p65*; The content of TLR4, NF-κB-p65, β-APP, PS-1	Lycopene significantly improves cognitive deficits by blocking the activation of NF-κB p65 and TLR4 expressions and the production of cytokines, thereby endorsing its usefulness for diminishing β-amyloid deposition in the hippocampus tissues.	[116]
	Crocin	Middle-aged (15 months old) rats	Serum parameters assay; SOD, CAT, GPX, GR, GST, Na^+^/K^+^ ATPase, Ca^2+^ ATPase, AChE, CS and CCOX activity; GSH, LPO, PCC, ROS, NO, LF and acetylcholine levels; Histopathology assay.	Oral supplementation of crocin reverses the aging of rats through the suppression of oxidative stress and neuroinflammatory responses.	[117]
Xanthophylls	Astaxanthin (ATX)	D-Gal-induced-aging rats	Activities of SOD, CAT and GPX; MDA content; Serum levels of IL-2, IL-1β, IL-6, IgM and IgG; Nrf2, Keap1, NF-κB (p65) and IκBα level.	The anti-aging effect of astaxanthin is in part due to Nrf2/Keap1 and NF-κB pathways, which regulate oxidative stress and immune impairment, respectively.	[118]
	ATX	Young and aged male mice	Level of MDA, NO, APOPs and GSH; CAT and SOD activity;	ATX alleviates brain aging by suppressing oxidative stress.	[119]
	ATX	Wild-type yeast *Saccharomyces cerevisiae* and antioxidant-deficient strains	Survivability assay; Antioxidant biomarkers assay; Apoptotic markers assay.	ATX enhances the longevity of yeast *S. cerevisiae* by reducing oxidative stress and apoptosis.	[120]
	ATX	Glutamate-treated mouse hippocampal HT22 cells	Cell viability assay; LDH activity assay; ROS content; Caspase activity and PARP cleavage assay; The relative mRNA expression of *HO-1*; The content of HO-1, Nrf2, p-Akt, Akt, GSK3b, p-GSK-3b (Ser9), Bcl-2, Bax, AIF, cytochrome-c (Cyto-c), PARP.	ATX alleviates glutamate excitotoxicity-related neuronal loss associated with Alzheimer’s disease by regulating oxidative stress and apoptosis through the Akt/glycogen synthase kinase-3b (GSK-3b) signaling pathway.	[121]
	Krill oil (rich in ATX)	Aged *N. guentheri*	Lifespan assay; Histological assay; LF content; SA-β-Gal activity; ROS, protein oxidation, lipid peroxidation and p66Shc level; CAT, SOD and GPX activity; The relative mRNA expression of *interferon-γ*, *tnf-α* and *il10*; The content of IκBα, p-IκBα, p65 and p-p65.	KO exerts its anti-aging and rejuvenation effects via the enhancement of the antioxidant system and suppression of the NF-κB signaling pathway.	[122]
	Canthaxanthin	Aging model liver cell (AML12) exposed to H_2_O_2_; Liver fibrosis mice model induced by CCL4	In-vitro study: SA-β-Gal activity; P16, P21, γ-H2A, 53BP1, HP1α and H3K9me3 levels; The relative mRNA expression of *IL-6*, *IL-1β*, *TNF-α*, *CXCL1* and *MMP-1*; Level of SIRT6, NF-κB, p-NF-κB, JAK2, p-JAK2, STAT1 and p-STAT1; Levels of ROS, MDA, SOD and GSH. In-vivo study: Histological analysis; Serum levels of ALT and AST; Levels of TNF-α, IL6, IL-1β, COL1a1 and α-SMA; Phosphorylation level of Smad2/3 protein.	Canthaxanthin alleviates liver aging and fibrosis by suppressing inflammation and oxidative stress, which it achieves by regulating SIRT6.	[123]
	Lutein	Fibroblasts exposed to UVA or UVB	Cell viability assay; Membrane integrity; MMP-1, MMP-2, TIMP-1, TIMP-2 and elastin protein levels; MMP-1 promoter activity.	Lutein alleviates fibroblast aging induced by UVA or UVB radiation through the inhibition of the MMP to TIMP ratio, cell loss, membrane damage and elastin expression.	[124]
	Lutein	Human RPE cell line ARPE-19 treated with H_2_O_2_	Cell viability assay; Lysosome and ROS content; SA-β-Gal activity; Cell cycle; The content of p-SIRT1, SIRT1, SIRT3, p-p53, p53, p21, HO-1, NQO1 and Nrf2.	Lutein protects cells from cellular senescence induced by oxidative stress by upregulating antioxidant effectors.	[125]

**Table 4:** Anti-aging effects and modes of action of sterols.

Subclasses of Sterols	Representative Components	Models	Main Indicators of Aging	Modes of Action	Reference
Phytosterols	Diosgenin (DG)	Aged *N. guentheri*	Lifespan; LF content; SA-β-Gal activity; ROS, protein oxidation and lipid peroxidation level; CAT, SOD and GPX activity; 20S proteasome activity; PI3K/AKT/mTOR activity; The relative mRNA expression of *pten*, *tsc1* and *tsc2*.	DG exerts its rejuvenation and anti-aging activity by inhibiting the PI3K/AKT/mTOR signal pathway and promoting the ubiquitin–proteasome pathway and antioxidant enzyme activity; they all play prominent roles in ROS production.	[138]
	β-Sitosterol	H_2_O_2_-treated HDF and HaCaT cells	Cytotoxicity of β-sitosterol; Hyaluronic acid (HA) content; Levels of hyaluronic acid synthases (HAS1, -2, -3) and hyaluronidases (HYAL1, -2, -3); Levels of aquaporin3 (AQP3), loricrin (LOR), filaggrin (FLG) and involucrin (IVL).	β-Sitosterol prevents skin aging by promoting the biosynthesis of HA and enhancing skin barrier function.	[139]
	Daucosterol palmitate (DSP) extracted from *Alpinia oxyphylla* Miq	Male AD rat model	Spatial learning and memory test; ROS level; Histological examination; Synaptophysin level.	DSP ameliorates Aβ-induced learning and memory impairment in rats by inhibiting ROS production, preventing hippocampal CA1 neuronal damage and restoring hippocampal synaptophysin.	[140]
	β-Sitosterol	Dexamethasone-induced muscle atrophy mice model and C2C12 myoblasts	Mice grip strength and treadmill analysis; Weight of muscles; Muscle fibers; Muscle tissue histological analysis; MAFbx, MuRF1, FoxO1 and FoxO3 level.	β-Sitosterol alleviates aging sarcopenia by downregulating transcriptional factor FoxO1, making FoxO1 unable to affect the expression of muscle atrophy F-box (MAFbx), ultimately inhibiting muscle atrophy.	[141]
	β-Sitosterol	TNF-α-treated GT1-7 cells (a cell line of GnRH neurons)	Membrane sterols test; Gonadotropin-releasing hormone (GnRH) release assay; Levels of NF-κB and p-IκBa.	β-Sitosterol prevents TNF-α-induced GnRH decline through the inhibition of NF-κB activation via the ER-mediated inhibition of IκB processing.	[142]
Animal sterols	Vitamin D3	D-Gal-induced-aging male rats	MDA content; SOD, GSH and CAT activity; GCNA and PCNA content; Anti-apoptotic (BCL2) and apoptotic (BAX and active caspase-3) assay; TUNEL immunohistochemical assay; Heat shock protein1 a1 (HSP1A1), AGE-receptor (AGER), vitamin D and advanced glycation end products (AGE) level.	Vitamin D3 improves age-associated spermatogenesis impairment by regulating apoptosis and the antioxidant system, which are involved in the AGER and HSP1A1 pathway.	[143]
	Vitamin D	Human lung carcinoma A549 cells, human B-cell lymphoblastoid TK6 cells, and human peripheral blood lymphocytes treated with H_2_O_2_	Detection of histone H2AX phosphorylation and ATM activation; ROS level; Analysis of cellular fluorescence.	Vitamin D exerts an anti-aging effect by attenuating DNA damage.	[144]
	Mussel (*Mytilidae*) sterols	Wild-type and mutant-type *Saccharomyces cerevisiae*	Lifespan assay; ROS level; MDA content; The relative mRNA expression of *uth1*, *skn7*, *tub1*.	The anti-aging effect of mussel sterols depends on their antioxidative ability and regulation of *uth1*, *skn7*, *tub1* expression.	[145]
Fungal sterols	Ergosterols from *Ganoderma lucidum* spores	Wild-type and mutant-type *Saccharomyces cerevisiae*	Replicative lifespan; The relative mRNA expression of *sod1* and *sod2*.	*Ganoderma lucidum* ergosterols prolong the replicative lifespan of yeast by regulating *uth1* expression.	[146]
	Chinese cordyceps cerevisterol	H9C2 cells; ICR mice	In-vitro study: Cell survival rate assay; Level of MAPK1, MAPK3, VEGFA, AKT1, PIK3CA and RAC1. In-vivo study: Angiogenic test.	Chinese cordyceps cerevisterol exhibits anti-aging and anti-fatigue effects by improving the body’s hypoxia tolerance through the VEGF signal pathway.	[147]

**Table 5:** Anti-aging effects and modes of action of terpenoids.

Subclasses of Terpenoids	Representative Components	Models	Main indicators of Aging	Modes of Action	Reference
Hemiterpene	Prenol	Wild-type and mutant-type *C. elegans*	Toxicity assessment; Lifespan assay; Amyloid-β-mediated paralysis, stress resistance and chemotaxis assays; Level of α-synuclein and ROS; The relative mRNA expression of *sod-1*, *sod-2*, *sod-3*, *gst-3*, *gst-4*, *hsp 16.2*, *hsp-70*, *daf-16*, *skn-1* and *hsf-1*.	Prenol improves the lifespan and health span of worms by regulating transcription factors DAF-16, HSF-1 and SKN-1.	[170]
Monoterpenoids	Cuminaldehyde extracted from cumin	SH-SY5Y cells; C57BL/6J male mice	In-vitro study: Cell survival assay. In-vivo study: Spatial learning and memory assay; The relative mRNA expression of *Bdnf*, *Icam*, *ApoE*, *TNF-α* and *IL-6*.	Cuminaldehyde exerts neuroprotective effects through the modulation of genes coding for neurotrophic factors and inflammatory factors.	[171]
	Asperuloside extracted from Du Zhong *Eucommia ulmoides* male flower	*C. elegans*	Motor competency assay; Mitochondrial respiratory capacity; ATP content; ROS level; DAF-16 level; Metabolomics detection; RNAi interference assay for *daf-16*.	Asperuloside delays the muscle aging of *C. elegans* through a *daf-16*-mediated improvement in mitochondrial dysfunction.	[172]
	Limonene	HaCaT cells treated with UVB	Cell viability assay; ROS level; HO-1, NQO-1 and γ-GCLC content; *Nrf2* knockdown assay; Levels of α-MSH, p53, claudin, occuludin, ZO-1 and MMP-2.	Limonene displays a dermato-protective effect in skin cells by activating the Nrf2-dependent cellular antioxidant defense system.	[173]
	Carvacrol (CAR)	D-Gal-induced-aging rats	TAC and GPX activity; GST level; MDA content; The relative mRNA expression of *p53*, *p21* and *Bax*.	CAR attenuates aging by suppressing oxidative stress.	[174]
Sesquiterpenoids	Dihydro-β-agarofuran-type sesquiterpenoid	*C. elegans*	Lifespan assay; The relative mRNA expression of *skn-1*, *hsf-1* and *daf-16*.	Dihydro-β-agarofuran-type sesquiterpenoids prolong the lifespan of worms by regulating transcription factors *skn-1* and *hsf-1*.	[175]
	α- and β-Santalol	Wild-type and mutant-type *C. elegans*	Lifespan assay; ROS level; Stress resistance assay; Toxic amyloid-β and polyglutamine repeat (Q35, Q40, and HtnQ150) aggregation assay; Neuronal survival assay; LF accumulation; RNAi interference assay for *skn-1* and *let-23*; The relative mRNA expression of *gst-4*, *gcs-1*, *gst-10*, *gsr-1*, *hsp-4* and *skr-5*.	Both α- and β-Santalol alleviate aging by selectively regulating SKN-1/Nrf2 and EOR-1/PLZF transcription factors through the RTK/Ras/MAPK-dependent signaling axis.	[176]
	Patchouli alcohol (PA) extracted from patchouli *Pogostemon cablin*	D-Gal-induced-aging mice; Chondrocytes isolated from D-Gal induced-aging mice	In-vitro study: Cell survival assay; The relative mRNA expression of *col2a1*, *mmp13*, *Tp53*, *CDKN1A*, *cat*, *gss*, *sod*, *Nrf2*, *HO-1*, *Keap1* and *NQO1*; Content of Acan, Col2a1, Adamts5, Mmp13, TP53 and P21. In-vivo study: Mental state, body weight and organ index assay; Histopathological assay; Cartilage quality assay; The relative mRNA expression of *Tp53*, *Cdkn1a/p21^Cip1/Waf1^*, *Cdkn2a/p16^INK4a^*, *Nrf2*, *HO-1*, *cat* and *sod*; CAT and SOD activity; GSH and MDA level.	PA inhibits D-Gal-induced chondrocyte senescence via the activation of the antioxidative system, which is attributable to the activation of the Nrf2/HO-1 pathway.	[177]
Diterpenoids	Mushroom *Hericium erinaceus* mycelium (HEM) and its diterpenoid derivative, erinacine A (EA)	15-month-old mice	Spatial learning ability assay; The mRNA expression of *TNFα*, *IL-1β*, *NGF* and *NeuN*; Body weight and fat pad weight assay; Serum chemistry analysis.	HEM and EA minimize the progression of aging and obesity-induced neurodegeneration by reducing metabolic abnormalities and neuroinflammatory cytokines and increasing neurogenesis factors.	[178]
	Carnosol extracted from *Rosmarinus*	Wild-type and mutant-type *C. elegans*	Lifespan assay; ROS level; CAT, SOD and GPX activity; MDA content; Stress resistance, mobility, fertility and paralysis assay; Age pigment and body fat accumulation; The relative mRNA expression of *sod-3*, *sod-5*, *hsf-1*, *hsp-16.1*, *hsp-16.2*, *daf-16* and *hsf-1*; Subcellular localization of DAF-16.	Carnosol-mediated longevity requires the upregulated expression of *sod-3*, *sod-5*, *hsf-1*, *hsp-16.1* and *hsp-16.2* and is dependent on *hsf-1* gene.	[179]
	Andrographolide (ANDRO) extracted from Chuan Xin Lian *Andrographis paniculata*	Adult and aged *Octodon degus*	Recognition memory, preference for novel experiences, social recognition and long-term memory assay; Level of glutamate ionotropic receptor AMPA type subunit 1 (GLUR-1), glycogen synthase kinase-3β (GSK-3β), phosphorylated glycogen synthase kinase-3β (p-GSK-3β), Homer, N-methyl D-aspartate receptor subtype 2B (NR2B), post-synaptic density 95 (PSD95), synaptotagmin I/II (SYT), synaptophysin (SYP) and gamma-aminobutyric acid receptor (GABA_A_).	ANDRO administration shows improved complex behaviors related to age-detrimental effects by modulating mechanisms of synaptic transmission and proteins.	[180]
Triterpenes	Compound K (CK) extracted from herb *Panax ginseng*	Hydrobromide-induced memory impairment mice	Production and clearance of Aβ; SOD, MDA, and GSH levels; Bcl-2, Bax and Caspase-3 levels; The relative mRNA expression of Nrf2 signaling pathway-related factors *Nrf2*, *Keap1* and *HO-1*.	CK improves impaired memory function by inhibiting apoptosis and enhancing stress resistance through the Nrf2/Keap1 signaling pathway.	[181]
	CK	Vascular-dementia (VD) rats	Cognitive ability assay; Histopathological alterations assay; The deposition of Aβ_1–42_; The contents of Ser473-Akt/Akt, pSer9-GSK3β/GSK3β and insulin degrading enzyme (IDE).	CK might attenuate cognitive deficits and Aβ_1–42_ deposition in the hippocampus by enhancing the expression of pSer9-GSK-3β and IDE.	[182]
	CK	Mouse embryonic fibroblast NIH3T3 cells treated with UVB	Cell viability assay; The mRNA expression levels of *MMP1*, *COX-2*, *filaggrin* (*FLG*), *transglutaminase* (*TGM*), *hyaluronic acid synthases* (*HAS*)-*1*, *2*, and *3*, *type I collagen*; Type I collagen promoter activity assay; Melanin formation and secretion assay; Tyrosinase activity assay; The content of κBα/p-κBα, and JNK/p-JNK, ERK/p-ERK, and p38/p-p38.	CK has ability to increase skin moisture levels and melanin synthesis as well as protect against UVB-induced photo-aging by regulating the p38/AP-1/CREB pathway.	[183]
	Ginsenoside Rg1 (Rg1)	Sca-1^+^ HSC/HPC cells harvested from D-Gal-induced-aging rats	SA-β-Gal activity; Cell-cycle assay; CFU-mix assay; The mRNA expression of *cleaved caspase 3*, *Bcl-2*, *Bax*, *SIRT3* and *SOD2*; The content of SIRT3 and SOD2.	Rg1 conducts functions of anti-aging in Sca-1^+^ HSC/HPC cells in D-gal-induced-aging rats by inhibiting mitochondrial pathway-mediated apoptosis and activating the SIRT3/SOD2 signaling pathway.	[184]
	Ginsenoside Rb1 (GRb1)	Young and natural aging C57BL/6J mice	Morphological and histological analysis; The relative mRNA expression of *PAI-1*, *Mmp12*, *Tert*, *p53*, *p21*, *Cdk2*, *Bax*, *Caspase-3*, *IL-1β*, *IL-6*, *IL-8* and *TNF-α*; The content of p53, p21, Cdk2, Bax, Cleaved caspase-3, NF-κB, p-NF-κB; Metabolomics analysis.	GRb1 retards the aging process in natural-aging C57BL/6J mice by regulating the cell cycle and apoptotic pathway, which are associated with the alleviation of metabolic disorders.	[185]
	20-O-β-D-glucopyranosyl20(S)-protopanaxadiol (20GPPD), the primary bioactive metabolite of ginsenoside Rb1	Human keratinocytes HaCaT	Cell viability assay; The content of HA and hyaluronan synthase 2 (HAS2); The level of ERK, Akt, p-ERK, p-Akt, Src and p-Src.	20GPPD enhances the production of HA by acting as an upstream modulator of ERK and Akt activity mediated by Src kinase in human keratinocytes.	[186]
	Ganoderic acid D (GA-D) extracted from *Ganoderma lucidum*	Human amniotic mesenchymal stem cell (hAMSC) treated with H_2_O_2_	SA-β-Gal activity; Telomerase activity assay; Cell viability assay; ROS level; Osteogenic and chondrogenic differentiation of hAMSCs assay; Cell-cycle assay; The relative mRNA expression of *14-3-3ζ*, *PRDX3*, *SIRT1-7*, *β-Catenin*, *ERK1*, *ERK2*; The content of p21, p16, Nrf2, PERK, p-PERK, and peroxidase III (PRDX3).	GA-D retards hAMSC senescence through the activation of the PERK/NRF2 signaling pathway.	[187]
	GA-D	hAMSCs treated with or without GA-D; Mice treated with or without GA-D	In-vitro study: 14-3-3ε-Encoding gene (*YWHAE*) knockdown assay; SA-β-Gal activity assay; Cell viability assay; ROS level; Differentiation of hAMSCs assay; Cell-cycle assay; The content of p21, p16, 14-3-3ζ, t-Nrf2, n-Nrf2, HO-1, NQO1, CaM, p-CaMKII, CaMKII, p16INK4a; The relative mRNA expression levels of *14-3-3ε*, *n-Nrf2*, *t-Nrf2*, *CaM*, *CaMKII*, *p-CaMKII*, *HO-1*, *NQO1*, *p16^INK4a^*, and *p21*; In-vivo study: Histopathological assay; Activity of T-AOC, SOD, GPX, and content of MDA, AGEs, RAGE assay in the sera.	GA-D retards hAMSC senescence by targeting 14-3-3ε to activate the CaM/CaMKII/Nrf2 signaling pathway.	[188]
	Maslinic acid (MA) obtained from *Olea europae*	Rats pretreated with CCl_4_	Plasma lipoperoxide levels and liver lipid peroxidation assay.	MA may offer some advantages in the anti-aging process by decreasing plasma lipoperoxide levels and inhibiting liver lipid peroxidation.	[189]
	Ursolic acid (UA)	Skeletal muscle satellite cells, isolated from 10-days old mice, treated with or without UA; 10 months aged mice treated with UA	In-vitro study: Cell viability assay; The relative mRNA levels of *paired-box 7* (*PAX7*), *Myogenin*, *peroxisome proliferator-activated receptor gamma* (*PGC-1α*) and *SIRT1*; Immunostaining assay. In-vivo study: The content of myoglobin; The concentration of ATP and ADP; Histopathological assay; Satellite cell proliferation assay.	UA promotes skeletal muscle rejuvenation by enhancing SIRT1 expression and satellite cell proliferation.	[190]
	Ursolic Acid (UA), a compound which is extensively present in apple peels	Aged-mice C5BL/6 (20 months old)	The content of SIRT1, SIRT6, PGC-1β and α-Klotho.	UA treatment reverses the aging of the liver by enhancing SIRT1 and SIRT6 levels and promoting the production of PGC-1β and Klotho.	[191]
	Cycloastragenol (CA)	*C. elegans*	Lifespan assay.	CA and its new derivatives could significantly extend the lifespan of *C. elegans* by regulating *SKN-1*.	[192]
	CA	Human T cells	Cellular proliferative capacity; Telomerase activity; Surface markers and cytokine secretion of human CD4^+^ and CD8^+^ T cells.	CA extends T cell proliferation by increasing telomerase activity.	[193]
	Platycodin D extracted from *Platycodon grandiflorum*	C2C12 cells treated with H_2_O_2_	Cell cytotoxicity assay; ROS level; The relative mRNA levels of *Bcl-2*, *Bax* and *caspase-3*; Level of Keap1, Nrf2 and HO-1.	Platycodin D protects C2C12 cells against H_2_O_2_-induced oxidative stress and apoptosis through the Keap1/Nrf2/HO-1 signal pathway.	[194]
	Ginsenoside Re (GRe)	4-month-old young male mice and 14-month-old aged male mice, including *Klotho* wild-type, *Klotho*-deficient (±) mice, wild-type, GPx-1 KO, non-transgenic (non-TG), and GPx-1 TG mice	Cognitive and memory ability assay; DNA-binding activity assay; The content of JAK2, p-JAK2, STAT3, p-STAT3, angiotensin II AT1 receptor, ET1, GPx-1, ERK, p-ERK, cAMP response element-binding protein (CREB), and p-CREB; NOX activity assay; ROS, lipid peroxidation and protein carbonyl level assay; The relative mRNA level of *glutamate-cysteine ligase catalytic subunit* (*GCLc*) and *glutamate-cysteine ligase modifier subunit* (*GCLm*); The level of GSH and GSSG; SOD, GPX and GR activity assay.	GRe attenuates all alterations, such as AT1 receptor expression, NOX, ROS, and GPX levels, and cognitive dysfunction in aged *Klotho*-deficient (±) mice via the upregulation of Nrf2/GPx1/ERK/CREB signaling.	[195]
	Ginsenoside Rg1 (Rg1)	Sca-1^+^ HSC/HPC cells isolated from D-Gal-induced-aging rats	SA-β-gal activity; Cell-cycle analysis; CFU-mix assay; The relative mRNA expression of *cleaved caspase 3*, *B-cell lymphoma-2* (*Bcl-2*), *Bcl-2 associated X protein* (*Bax*), *SIRT3*, *SOD2*; The content of SIRT3 and SOD2.	Rg1 conducts functions of anti-aging in Sca-1^+^ HSC/HPC cells in the D-gal-induced-aging model by inhibiting mitochondrial pathway-mediated apoptosis and activating the SIRT3/SOD2 signaling pathway.	[184]
	Rg1	SAMP8 mice	ROS level; The relative mRNA expression of *NOX4*, *p22phox* and *p47phox*; Pathological examination; Level of collagen IV, TGF-β and NLRP3.	Rg1 reduces age-related liver fibrosis by reducing NOX4-mediated ROS-induced oxidative stress and inhibiting the activation of the NLRP3 inflammasome.	[196]
	Rg1	AlCl3-induced-aging mice	Spatial learning and memory assay; Level of BDNF, TrkB, FGF2, Akt, Bcl-2 and Caspase3.	Rg1 ameliorates cognitive deficits in aging mice by regulating FGF2-Akt and BDNF-TrkB signaling pathways.	[197]

**Table 6:** Anti-aging effects and modes of action of vitamins.

Subclasses of Vitamins	Representative Components	Models	Main Indicators of Aging	Modes of Action	Reference
Water-soluble vitamins	Riboflavin (RF)	*D. melanogaster* treated with H_2_O_2_	Lifespan assay; Reproductive capacity assay; LF content; Activities of SOD and CAT.	RF prolongs the lifespan and increases the reproduction of fruit flies through the antioxidative stress pathway.	[206]
	Topical liquid formula of polydeoxyribonucleotide (PDRN), vitamin C and niacinamide (a kind of vitamin B3 derivant)	Human primary epidermal keratinocyte, human primary epidermal melanocyte, human fibroblast treated with UV-B; HRM-2 mice treated with UV-B	Melanin content; Contents of NF-κB, Nrf, p-Nrf, HO-1, P53, MITF, Col1a1, Fibrillin1, Fibrillin2, Fibrillin5; mRNA expression of *p53*, *mitf*, *Mmp2*, *Mmp3*, *Mmp9*; Activity of NADPH oxidase and SOD.	A topical liquid formula delays skin-aging induced by UV-B by regulating Nrf2 signaling.	[207]
	Combination of vitamin C, rice and lupin bio-peptides, hyaluronic acid, and Vichy volcanic mineralizing water	Human keratinocytes and fibroblasts treated with UV-A and pollution	Anti-general oxidant, anti-lipid peroxidation and anti-protein glycosylation ability assays; Activity of collagenase, elastase and hyaluronidase.	A combination of vitamin C, rice and lupin bio-peptides, hyaluronic acid, and Vichy volcanic mineralizing water shows high global antioxidant capacity as well as a protective effect against oxidative stress induced by UV-A, pollution, or both combined factors by stimulating collagen synthesis.	[208]
	Vitamin C	Zebrafish treated with LiCl	Expressions of *GSK-3β*, *TSC2*, *mTOR*, *FAS*, *ACC*, and *ACL*; Content of TC, TG and NEFA.	Vitamin C delays liver aging by reducing lipid deposition by regulating GSK-3β/mTOR signaling.	[209]
Fat-soluble vitamins	Vitamin E	DNA repair-deficient mutant mouse (Xpg^−/−^)	Body weight and tremors assay; SS/SH redox ratio assay; Protein oxidation assay; Histological assay; p53-positive cells assay.	Vitamin E supplementation exerts obvious neuroprotective effect by reducing the number of p53-positive cells.	[210]
	Vitamin E; Velvet antler polypeptide	D-gal-induced-aging mice	Learning and cognitive abilities assay; Activities of SOD, GPX, and CAT; MDA, PPARα, ACOX1, CPT1A and APOE4 contents; Intestinal microecological analysis.	Vitamin E, or velvet antler polypeptide, exerts anti-aging activity by modulating the gut microbiota and regulating the PPARα/APOE4 pathway.	[211]
	Vitamin E and quercetin	Aged breeder hens	Intestinal morphology assay; Serum D-lactate and diamine oxidase content assay; Secretory immunoglobulin A level assay; Expression of *Mucin-2*, *occludin*, *ZO1*, *claudin-1*, *TNF-α*, *IL-6*, *IL-1β*, *IL-10*, *IL-4*, *Bax*, *Bcl-2*, *SOD1* and *GPx-2*. The levels of SOD, GPX, CAT and MDA. Total antioxidant capacity assay.	Quercetin and vitamin E improve intestinal function in aged breeder hens by protecting intestinal structure and integrity.	[212]
	Vitamin K and vitamin D	Human osteoblasts cultured in presence of hydroxyapatite-based biomaterials	Cell viability assay; ROS and GSH level assay; GPX and ALP activity; DNA proliferation assay.	Vitamins D and K protect redox balance and support growth of osteoblasts affected by hydroxyapatite-based biomaterials due to antioxidant properties.	[213]
	Vitamin K2	Wild-type and mutant-type *C. elegans*	Longevity and survival assay; RNA-sequencing analysis; Progeny and reproductive activity assay; Expression of *daf-12*, *fard-1*, *fat-5/6/7*, and *lipl-4*.	Vitamin K2 extends the lifespan of *C. elegans* and improves the resistance to pathogen infection, heat stress and H_2_O_2_-induced inner oxidative stress by enhancing fat metabolism.	[214]

## Data Availability

Data will be made available on request.
